# Diabetes and the Metabolic Syndrome as Drivers of Neurodegeneration: Convergent Mechanisms Linking Peripheral Neuropathy and Dementia

**DOI:** 10.1002/ana.78289

**Published:** 2026-07-07

**Authors:** Masha G. Savelieff, David L. Bennett, Troels S. Jensen, Eva L. Feldman

**Affiliations:** ^1^ Department of Biomedical Sciences University of North Dakota Grand Forks ND USA; ^2^ Nuffield Department of Clinical Neuroscience University of Oxford Oxford UK; ^3^ Department of Neurology Aarhus University Hospital Aarhus Denmark; ^4^ Danish Pain Research Center, Aarhus University Aarhus Denmark; ^5^ Department of Neurology University of Michigan Ann Arbor MI USA; ^6^ NeuroNetwork for Emerging Therapies, University of Michigan Ann Arbor MI USA

## Abstract

The metabolic syndrome, a state of progressive metabolic dysfunction, injures the peripheral and central nervous systems, promoting peripheral neuropathy (PN) and cognitive impairment (CI), respectively. We posit PN and CI are connected in the metabolic syndrome framework, built on the premise that neurons, whether in the peripheral or central nervous systems, are susceptible to similar injury from shared metabolic risk factors and pathophysiological processes. We highlight future studies for determining the relative evolution of PN and CI in metabolic syndrome, and propose revising the “stocking–glove” description of PN to “stocking–glove–hat” encompassing CI, concluding with research, therapeutic, and clinical implications. ANN NEUROL 2026;100:458–476

## Graphical abstract

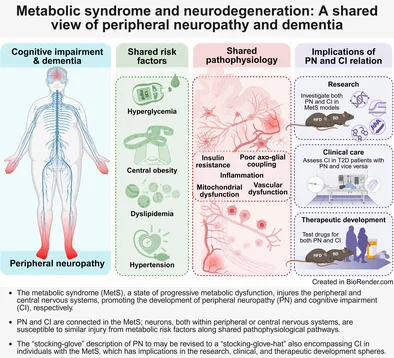

Diabetes is long associated with a suite of neurological complications. Prevalent among them is peripheral neuropathy (PN), a microvascular diabetic complication of the peripheral nervous system (PNS).^1^ Dementia, also, is increasingly and more definitively recognized as a diabetes complication, most recently articulated by the 2024 report of the Lancet standing Commission on dementia prevention, intervention, and care (referred to as the Lancet Commission hereon).^2^ Neurological injury secondary to metabolic dysfunction occurs early in the disease course; individuals with obesity and/or prediabetes, before the development of frank type 2 diabetes (T2D), are already at risk of PN^1^ and cognitive impairment (CI).^2^

This review outlines the clinical evidence that the metabolic syndrome (MetS), a state of metabolic dysfunction encompassing a suite of disorders, including either prediabetes or T2D, hyperlipidemia, hypertension, and obesity, underlies neurodegeneration throughout the nervous system, which presents clinically at variable stages as PN and dementia. Within this framework, the classical “stocking–glove” description of PN is reformulated as a “stocking–glove–hat,” reflecting the MetS‐induced neurodegenerative process in the PNS and central nervous system (CNS). We provide an overview of clinical studies that suggest that PN might correlate with dementia, and brain structural and functional changes in individuals with the MetS, asking, “Can I look at your feet and tell you about your brain?” Finally, the potential implications of an interconnection between the MetS‐acquired PN and dementia are described in the research, therapeutic development, and clinical care spheres.

## PN: The Stocking–Glove

Metabolically‐acquired PN usually occurs as symmetrical, length‐dependent nerve injury that mirrors the MetS patients’ symptomatology, encompassing numbness, tingling, and, for some patients, pain.^1^, ^3^ Damage first occurs distally in the feet, and progresses proximally; upon reaching the calves, injury initiates in the hands. This pattern is frequently referred to as “stocking–glove,” although the origin of this term is lost to time. Recent evidence potentially suggests an actual loss of PNS dorsal root ganglia (DRG) neurons,^4^ beyond only length‐dependent axonal loss that is more akin to neuronal loss in dementia. PN is highly prevalent, and can occur in up to 50% of type 1 diabetes (T1D) and of T2D patients, depending on age, disease duration, and diagnostic criteria.^3^ Peripheral nerve damage can also occur independent of diabetes, and is present in ≥10% of patients with prediabetes, a state of elevated fasting glucose and glycated hemoglobin (HbA1c) not meeting the formal criteria of diabetes,^5^ and 12% of normoglycemic obese individuals.^6^ The global burden of PN secondary to diabetes is massive,^7^ with a prevalence of 206 million cases globally in 2021, and an age‐standardized rate of 301.9 per 100,000 people. PN was the third leading cause of diabetes disability‐adjusted life‐years in adults aged 20–79 years.^7^ Besides the personal cost to patients from reduced quality‐of‐life and disability, PN also incurs substantial economic costs.^3^

## Dementia: The Hat

CI is defined as poor memory and an inability to concentrate, learn new information, or carry out everyday activities. CI rests on a spectrum, ranging from more subtle cognitive dysfunction not meeting formal neuropsychological criteria for a clinical CI entity,^8^ to mild CI (MCI), to the more progressed dementias, including Alzheimer's disease (AD) and AD‐related dementias (also encompassing vascular dementia [VaD]). In this review, we use the term CI as an umbrella designation for the entire spectrum or specify dementia when studies examined a specific clinical entity.

The global burden of dementia is very substantial,^7^ with a prevalence of 56.9 million cases globally in 2021 and an age‐standardized rate of 694.0 per 100,000 people,^7^ projected to rise to an estimated 150 million by 2050.^9^ AD and other dementias were the second leading cause of disability‐adjusted life‐years in adults aged 60–79 years and aged ≥80 years.^7^ Importantly, the studies did not account for dementia arising specifically secondary to diabetes; however, recognizing the contribution from diabetes and other metabolic dysfunction, the Global Burden of Diseases Dementia Forecasting Collaborators estimated the anticipated increase in dementia prevalence by 2050 arising from these modifiable risk factors, including elevated fasting glucose, obesity, dyslipidemia, hypertension, and physical inactivity, among others.^9^

## PN and CI Share Metabolic Risk Factors

Earlier studies of metabolically‐acquired PN and CI primarily focused on diabetes as the greatest risk factor; however, increasingly, evidence points to multifactorial risk factors from the MetS and its components. The MetS is a constellation of metabolic perturbations, generally resulting from unhealthy diets and sedentary lifestyles.^10^ The MetS encompasses elevated prediabetes and diabetes; that is, elevated fasting glucose, central obesity, dyslipidemia, and hypertension. The MetS is clinically defined as at least 3 of 5 metabolic impairments that fall outside normal ranges (Box 1).^12^

**FIGURE 1:**
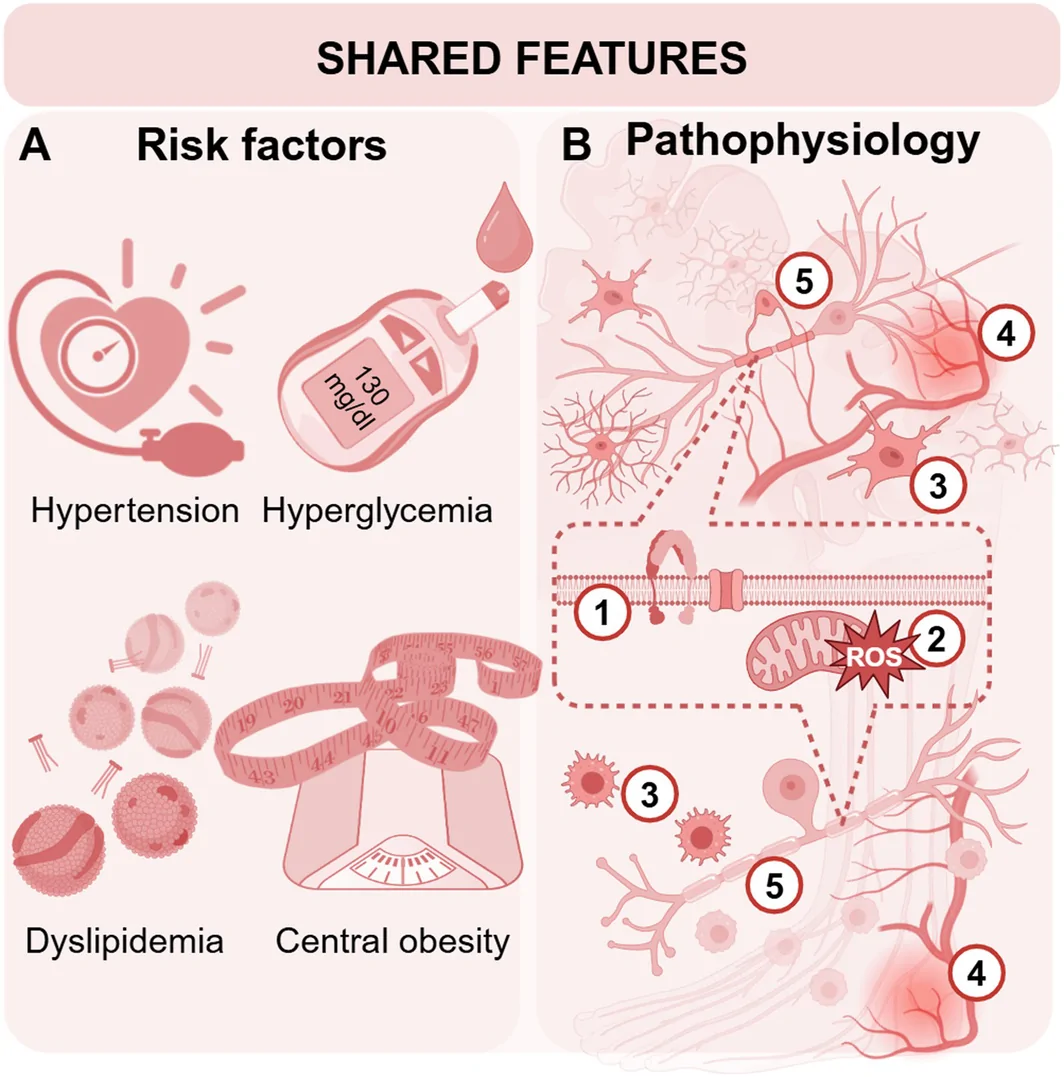
Shared features of metabolic syndrome‐related peripheral neuropathy and cognitive impairment. (A) Peripheral neuropathy and cognitive impairment share metabolic syndrome risk factors. (B) Peripheral neuropathy (lower) and cognitive impairment (upper) share metabolic syndrome‐induced pathophysiological pathways, spanning (1) insulin resistance and altered metabolism, (2) impaired mitochondrial dynamics and reactive oxygen species, (3) inflammation and immune dyshomeostasis, (4) vascular damage and impaired neurovascular coupling (axon reflex vasodilation in the peripheral nervous system), and (5) impaired axo–glial interactions. ROS = reactive oxygen species. Created in BioRender.com. [Color figure can be viewed at www.annalsofneurology.org]

In this review, we use the term MetS as an umbrella designation for formal MetS meeting the MetS criteria, as well as its various components, usually investigated in studies in the context of T2D, obesity, or comorbid T2D and obesity. Additionally, this review also considers studies in patients with T1D, who, through hyperglycemia, are at risk of and develop PN and CI, but also increasingly develop obesity and the MetS,^13^ contributing to neuropathy risk.^14^, ^15^ Overall, as recent studies demonstrate, both PN and CI share these metabolic risk factors (Fig 1A).

BOX 1 **Metabolic syndrome criteria**

Persons with the MetS meet ≥3 out of the 5 criteria below, based on the American Heart Association/National Heart, Lung, and Blood Institute Scientific Statement:^12^

- Raised waist circumference (≥88 cm women, ≥102 cm men)*
- Raised fasting glucose (>100 mg/dL) or on relevant medication
- Raised triglycerides (≥150 mg/dL) or on relevant medication
- Lowered high‐density lipoprotein cholesterol (<50 mg/dL women, <40 mg/dL men) or on relevant medication
- Raised systolic (≥130 mmHg) or diastolic (≥85 mmHg) blood pressure or on relevant medication in a patient with a history of hypertension

*Cutoffs are population‐dependent based on ancestral descent and issuing organization.^11^

### Diabetes is a PN and CI Risk Factor

Diabetes^16^ is the primary risk factor for metabolically‐acquired PN,^1^ bolstered by an extensive and definitive body of literature in large cohorts and population‐based studies, such as the Anglo‐Danish‐Dutch Study of Intensive Treatment in People with Screen‐Detected Diabetes in Primary Care (ADDITION),^17^ Danish Center for Strategic Research in Type 2 Diabetes (DD2),^18^ the Search for Diabetes in Youth (SEARCH),^14^ Treatment Options for Type 2 Diabetes in Adolescents and Youth 2 study,^19^ and Diabetes Control and Complications Trial/Epidemiology of Diabetes Interventions and Complications (DCCT/EDIC).^15^ Poor glycemic metrics enhance the risk of developing PN, including longer diabetes duration,^14^, ^15^ elevated HbA1c,^15^, ^17^, ^18^, ^19^ and steeper HbA1c changes.^17^ Furthermore, prediabetes might also potentially increase the odds of developing PN,^5^ especially small fiber neuropathy, although this might explained by comorbid risk factors;^20^ for example, obesity.

Diabetes is likewise a long‐known risk factor for CI and dementia,^8^ evidence also accrued from large cohorts and population‐based studies, including UK Whitehall II,^21^ UK Biobank,^22^ Dutch Maastricht Aging Study,^23^ and US Atherosclerosis Risk in Communities (ARIC) study.^24^ The Lancet Commission now definitively recognizes midlife diabetes as a dementia risk factor,^2^ with a relative risk of 1.7^25^ and weighted population attributable fraction of 2.3%.^2^ Prediabetes might also be linked to CI, as shown in the ARIC study,^26^ although this risk might be explained by the subsequent T2D development.

### Obesity is a PN and CI Risk Factor

Although T2D is a long‐known and prominent neuropathy risk factor, strict glycemic control does not fully prevent PN.^27^ A growing number of studies show that the MetS component, obesity, even independent of glycemic status, contributes to PN. ADDITION identified general obesity by body mass index (BMI) and central obesity by waist circumference (WC) as incident PN risk factors independent of diabetes at 13‐year follow up.^28^ DD2 recapitulated these findings, with BMI and WC linked to PN prevalence ratio, adjusted for age, sex, and even diabetes duration.^18^ In our clinical Michigan cohort, we similarly found that WC, but not BMI, raised the odds of PN in fully adjusted analyses.^29^ In American Indians, we found that an increasing number of the MetS components tracked with faster PN progression.^30^ Overall, obesity as a PN risk factor has become well‐established,^1^ and the American Diabetes Association now recommends exercise and weight loss in addition to glucose control for PN prevention or onset delay.^31^

The scenario is mirrored by obesity as a CI risk factor; meta‐analysis of 14 studies encompassing 77,890 participants found that high midlife BMI enhanced the risk of all‐cause dementia, AD, and VaD.^32^ Meta‐analysis of 21 studies of 5,060,687 participants found that central obesity by WC also raised the risk of CI and dementia.^33^ In our work, we found that larger WC was linked to poorer cognitive performance based on a composite score from a battery of tests, adjusted for age, premorbid cognitive function, and education level.^6^ The Lancet Commission now also lists midlife obesity as a dementia risk factor,^2^ with a relative risk of 1.3^32^ and weighted population attributable fraction of 1.4%.^2^

### Dyslipidemia is a PN and CI Risk Factor

Dyslipidemia, defined as an abnormal blood lipid profile in triglycerides, low‐density lipoprotein cholesterol (LDL‐c), and high‐density lipoprotein cholesterol (HDL‐c) levels, is another prominent component of the MetS, and has been implicated as a risk factor for PN. In the ADDITION study, lower baseline HDL‐c and LDL‐c were associated with incident PN at follow up; however, analyses only adjusted for age, sex, and trial randomization.^28^ In contrast, DD2^18^ and DCCT/EDIC^15^ linked reduced HDL‐c to higher PN prevalence ratio or risk after adjusting for diabetes duration or HbA1c, as well as age. Hypertriglyceridemia is also variably linked to PN in some^15^, ^18^, ^29^ but not other^17^, ^28^ studies. Overall, dyslipidemia may influence PN risk, although more definitive studies are needed.

Regarding dyslipidemia and CI, an earlier 2020 Lancet Commission report stated there was insufficient evidence for LDL‐c as a dementia risk factor.^34^ Re‐analysis of the literature landscape in 2024 concluded that high midlife LDL‐c was a dementia risk factor based on large, recently published studies. Meta‐analysis of 17 studies involving 1.2 million participants found that elevated LDL‐c increased all‐cause dementia incidence,^35^ results echoed by analysis of the massive UK Clinical Practice Research Datalink database.^36^ Similarly, in a relatively small study (n = 4,932), higher HDL‐c protected against AD in age‐, sex‐, and education‐adjusted models.^37^ Presently, the Lancet Commission ascribes a relative risk of 1.3 to high LDL‐c,^38^ with a weighted population attributable fraction of 6.9%,^2^ higher than either T2D and obesity due to widespread prevalence of elevated LDL‐c.

### Hypertension is a PN and CI Risk Factor

Finally, the last MetS component, hypertension, is also a potential contender as a PN risk factor. DD2 found that antihypertension drug use, but, surprisingly, neither systolic nor diastolic blood pressure (BP), was linked to PN in T2D.^18^ ADDITION similarly found no correlation of systolic or diastolic BP to incident PN in T2D.^17^, ^28^ However, DCCT/EDIC identified both systolic and diastolic BP as a PN risk factor in T1D.^15^ PN risk was also associated with systolic BP in individuals with severe obesity in adjusted analyses in our Michigan cohort^29^ and in a systematic meta‐analysis of T2D studies.^39^ In a similar vein, the Lancet Commission recognizes midlife hypertension as a dementia risk factor, with a relative risk of 1.2^40^ and weighted population attributable fraction of 2.2%.^2^

Overall, the cumulated evidence suggests that PN and CI share several metabolic risk factors and occur in individuals with the MetS. Risk factors might also be mechanistically linked to the ensuing pathophysiology in PN and CI, as discussed in the following section, although the specific pathways are not yet fully elucidated.

## MetS‐Induced PN and CI Share Pathophysiology

It is long‐established that diabetes damages blood vessels, giving rise to the classical categorization of its complications as microvascular (affecting small vessels) or macrovascular (affecting large vessels); diverse mechanisms contribute, including advanced glycation end‐products formation,^41^ accelerated atherosclerosis and proteinaceous buildup, and elevated reactive oxygen species formation along with oxidized lipotoxic species.^42^ Although PN is a diabetic microvascular complication, its pathophysiology is multifactorial, extending beyond vascular dysfunction.^43^ Dementia in diabetes is also characterized by small vessel injury, promoting the development of white matter hyperintensities, lacunae, and microbleeds,^8^, ^44^ mixed with a complex AD‐like dementia with inflammatory and metabolic components.

**FIGURE 2:**
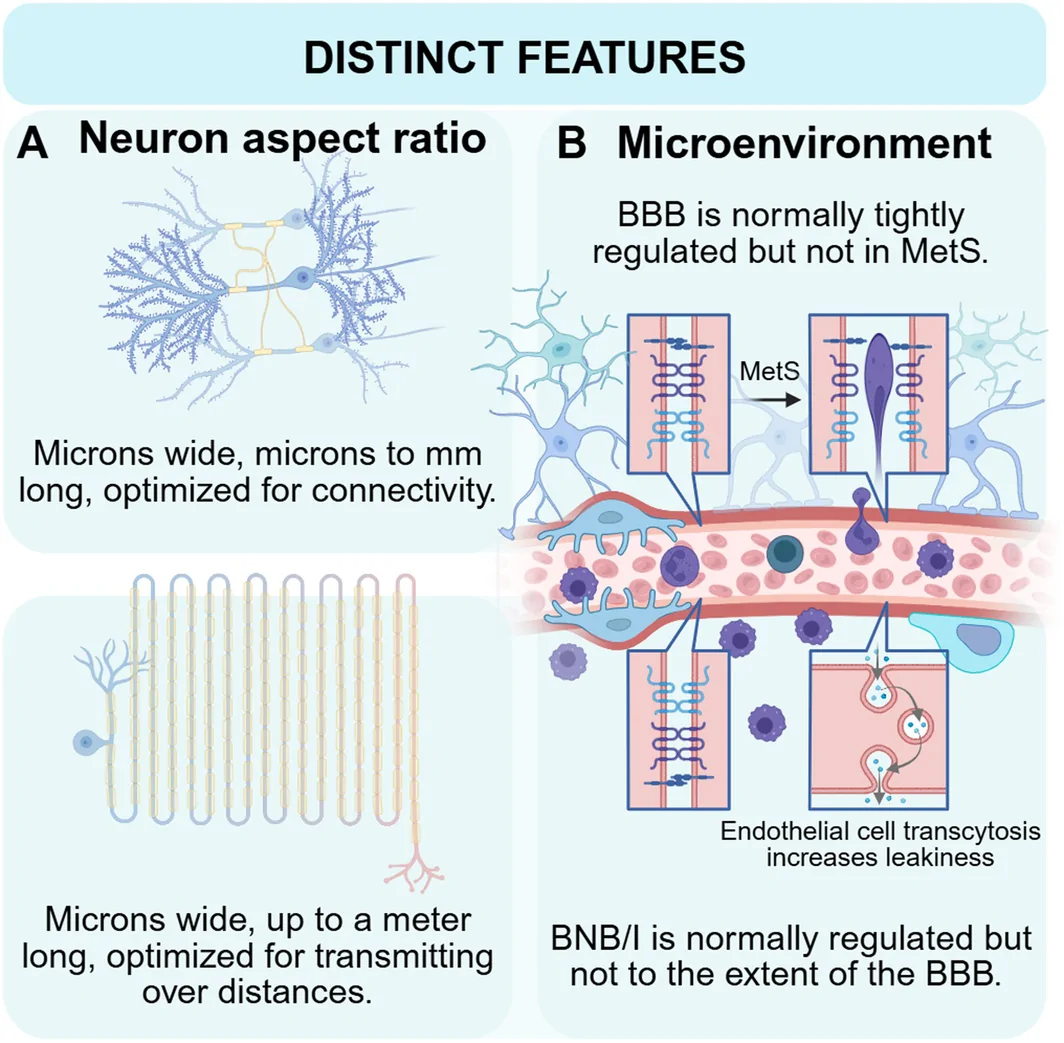
Distinct features of metabolic syndrome‐related peripheral neuropathy and cognitive impairment. (A) Peripheral neurons (lower) have much larger aspect ratios ≥50,000:1; that is, proportionately much longer compared with their width than central neurons (upper; up to 1,000:1 aspect ratios), affecting energy distribution and cellular processes along axons, and rendering the most distal portions of peripheral nerves vulnerable during bioenergetic crises. Peripheral neurons are optimized to transmit electrical impulses across a large distance, whereas central neurons are highly branched, with numerous dendrites and dendritic spines, for extensive connectivity. (B) During homeostasis, the blood–brain barrier (upper) tightly regulates entry of molecules and cells from circulation into the brain. The blood‐peripheral nerve barrier or interface (BNB/BNI; bottom)^49^ similarly controls entry of circulating molecules and cells into the peripheral nerves but is less stringent than the BBB. The MetS causes a breakdown in both the blood‐brain ^44^ and ‐peripheral nerve barriers.^50^ BBB = blood–brain barrier; BNB = blood–peripheral nerve barrier; BNI = blood–peripheral nerve interface; MetS = metabolic syndrome. Created in BioRender.com. [Color figure can be viewed at www.annalsofneurology.org]

A comprehensive review of these pathways is outside the present scope (see Eid et al^43^ [PN] and Biessels et al^8^ [CI] for extensive reviews). However, the ensuing sections outline some shared pathways, drawing parallels between MetS‐induced PN and CI (Fig 1B), from animal models of obesity and T2D, which develop both PN^45^, ^46^ and CI.^47^, ^48^ We hypothesize that metabolic risk factors that injure neurons do so regardless of whether they reside in the PNS or CNS, and underpin the complex, multifactorial, and shared pathophysiology of PN and CI, respectively. Concurrently, differences in PNS and CNS neurons, such as different aspect ratios (Fig 2A) and protection from systemic metabolic insults (Fig 2B), might impact the relative temporal onset of PN and CI.

### Insulin Resistance and Altered Metabolism in MetS PN and CI

Besides its metabolism‐regulating activities, insulin also exerts pleiotropic neuromodulatory and neurotrophic effects in the PNS (reviewed by Grote et al^51^) and CNS (reviewed by Arnold et al),^52^ promoting neurogenesis and repair, and protecting against oxidative stress and apoptosis, which enhance peripheral nerve activity and brain health, cognition, and memory formation. Insulin signaling also regulates food intake, which in turn impacts weight and systemic metabolism.^53^ Although PNS and CNS neurons take up glucose through insulin‐independent glucose transporter 3, they can still develop insulin resistance concurrent with systemic insulin resistance that occurs in the MetS and/or T2D and obesity. Additionally, the supportive glia and other PNS and CNS tissues can also develop insulin resistance.^51^, ^52^ Together, neuronal and glial insulin resistance disrupts regular biochemical and cellular processes in peripheral nerves and the brain, interfering with neuronal activity. These insulin resistance‐perturbed pathways include altered neural metabolism, mitochondrial dysfunction, impaired neurotransmitters, and inflammation in both the PNS^51^ and CNS,^52^ leading to neurodegeneration. Thus, peripheral nerve^51^ and hippocampal insulin resistance^54^ are potential contributors to PN and CI, respectively, in the context of the MetS.

### Impaired Mitochondrial Dynamics in MetS PN and CI

Besides the effects of insulin resistance on PNS and CNS tissues, hyperglycemia and dyslipidemia, both MetS components, perturb neuronal mitochondrial dynamics, including bioenergetics, trafficking, and biogenesis. Seamless bioenergetics is especially essential to neural tissues; the brain constitutes only 2% of body mass, but expends approximately 20% of resting metabolic energy.^55^ Likewise, PNS neurons spend massive amounts of energy to maintain a membrane potential across a vast surface area,^56^ and traffic mitochondria to areas up to 1 m distal to the cell body.^43^ Neuronal mitochondria occupy >50% of cytoplasm volume,^57^ far exceeding other cell types. To survive, neurons must coordinate energy production to meet high demands imposed by their complex morphology, rendering them particularly susceptible to metabolic insults that impair mitochondrial dynamics and bioenergetics, such as hyperglycemia and dyslipidemia.

In PNS DRG, hyperglycemia inhibits mitochondrial respiration and adenosine triphosphate production,^58^ increasing reactive oxygen species production and oxidative stress,^59^ and exacerbating insulin resistance. Dyslipidemia disrupts mitochondrial trafficking along axons, and alters their morphology and size.^58^, ^60^ In vivo, in obese, prediabetic high‐fat diet mice, mitochondria are depolarized, and fail to meet the energy demands of conduction at physiological frequencies.^61^ Sural nerve tissue from high‐fat diet mice analyzed ex vivo have diminished adenosine triphosphate production compared with proximal DRG, suggesting a bioenergetics crisis in the distal nerve.^62^

In CNS hippocampal neurons, hyperglycemia and dyslipidemia also disrupt mitochondrial function,^63^ and increase insulin resistance^64^ both in vitro^63^, ^64^ and in vivo.^64^ Hyperglycemia impairs axonal transport dynamics, and the machinery required to move mitochondria to areas of high energy demand in hippocampal neurons^65^ and in cerebral cortex.^66^ Dyslipidemia disrupts mitochondrial function^67^ and reduces cellular adenosine triphosphate levels,^68^ increasing reactive oxygen species generation^67^, ^68^ across multiple neuronal models.

### Inflammation and Immune Dyshomeostasis in MetS PN and CI

Inflammation and altered immune characteristics are additional shared features of MetS‐induced PN (reviewed by Eid et al^43^) and CI (reviewed by Heneka et al^69^ and Henn et al^70^). Under homeostasis, PNS‐resident macrophages locally surveil the endoneurial milieu and promote repair following injury.^43^ In MetS sciatic nerve, resident macrophages express elevated chemokine levels, while peripheral blood macrophages infiltrate before axonal loss or demyelination and might be protective, as ablating them accelerates PN.^71^ Sciatic nerve macrophages might adopt a more proinflammatory phenotype with more progressive disease, contributing to PN.^72^ In the CNS, microglia, the resident macrophages of the brain, surveil their surroundings and participate in housekeeping activities, such as synaptic remodeling and clearing debris.^69^, ^70^ Pathological aging in dementia triggers an innate microglial response, which fails to resolve, segueing into chronic inflammation with consequent neurodegeneration.^69^ In the MetS,^70^ activated hippocampal microglia contribute to high‐fat diet‐induced CI by phagocytic dendritic stripping,^73^ enhancing neuroinflammation in parallel with increased immune cell trafficking from the periphery into the CNS.^44^

### Vascular Damage and Impaired Neurovascular Coupling in MetS PN and CI

Vascular injury in the PNS is the pathology that lends PN its name as a microvascular complication in diabetes (reviewed by Eid et al^43^). Endothelial dysfunction, endoneurial capillary anomalies, basement membrane thickening, poor endoneurial perfusion, and ischemia are all present in PN secondary to diabetes, along with a suite of molecular changes, including diminished neurotrophic and angiogenic factor levels, and oxidative stress.^43^ A hyperglycemia‐induced drop in sciatic nerve blood flow promotes endoneurial hypoxia and decreases nerve conduction velocity.^74^ Like the PNS, the CNS in the MetS is characterized by endothelial dysfunction and stiffening, reduced vasoreactivity, impaired cerebral blood flow,^75^ chronic cerebral hypoperfusion, basement membrane thickening, increased capillary permeability,^44^ and a disrupted blood–brain barrier, along with molecular changes, such as oxidative and nitrosative stress (reviewed by van Sloten et al^44^). The concept of neurovascular coupling between CNS neurons and the brain vascular system is much more explored than in the PNS, but highlights the same intimate relationship between vascular factors and brain neurons.

### Impaired Axo–Glial Interactions in MetS PN and CI

Neurons do not function autonomously, but rely on a cadre of supporting glia. In the PNS, myelin‐forming Schwann cells also metabolically support axons by transferring energy metabolites, such as lactate, during neuronal activity^76^ and repair,^77^ whereas satellite glial cells transfer mitochondria to protect neurons from neuropathy.^78^ Impairment in these activities triggers neurodegeneration (reviewed by Eid et al^43^). The MetS conditions alter myelin lipid composition,^79^ induce peripheral nerve^51^ and Schwann cell^80^ insulin resistance, and promote Schwann cell apoptosis,^81^ which would interfere with energy support to axons, promoting nerve injury and PN. Several animal models with Schwann cell‐specific knockout of metabolic and mitochondrial genes show neuropathy features characteristic of PN.^43^

CNS oligodendrocytes, besides their myelinating activities, also metabolically support neurons by transferring energy substrates; for example, lactate,^70^ to enhance axonal bioenergetics and energy production^82^ during periods of activity^83^ (reviewed by Henn et al^70^). Abrogating oligodendrocyte monocarboxylate transporters to axons; that is, lactate transporters, promotes late‐onset hypomyelination and axonal degeneration.^84^ The MetS conditions modify myelin lipid composition^79^ and promote white matter loss.^85^ Furthermore, astrocytes can become insulin resistant, interfering with their role in recycling neurotransmitters.^52^

## Can I Look at Your Feet and Tell You About Your Brain?

As the MetS promotes neurodegeneration in both the PNS and CNS, the traditional “stocking–glove” description of PN is incomplete. We propose expanding the description to the “stocking–glove–hat” model, which incorporates both PN and CI/dementia. This new terminology more accurately describes the full spectrum of the neurological signs and symptoms present in patients with the MetS. Several center‐ and population‐based studies support this concept.

### PN Correlates to CI in the MetS

A growing literature showcases the presence of both PN and CI in patients with the MetS and/or T2D and obesity across various populations, suggesting generalizability (Table 1, Supplementary Table S1). In a large US cohort of T2D participants, the presence of PN correlated to various cognitive outcomes in highly adjusted analyses that accounted for potential confounding variables,^86^ mediated by larger WC; that is, central obesity. This relationship upholds for T1D; in a Chinese T1D case–control cohort^87^ and a German mixed T1D and T2D cohort,^88^ PN associated with CI by the Montreal Cognitive Assessment. In contrast, the Mini‐Mental State Examination might not be a sufficiently sensitive test of CI on PN populations.^89^, ^90^ Even independent of diabetes status, in a large US cohort of older adults, sensory PN correlated to incident dementia at 11‐year follow up in highly adjusted analyses.^91^

**TABLE 1:** Summary of Studies of Peripheral Neuropathy (PN) or Diabetic Peripheral Neuropathy (PN) to Cognitive Impairment or Dementia in Patients with or Without Diabetes

Study location, participants, design	Main study findings
Barzilay et al.;^86^ USA; T2D participants (n = 5,047); cross‐sectional, multi‐center cohort.	PN linked to CI outcomes, adjusted for age, sex, race, diabetes duration, WC, hyperlipidemia, hypertension, stroke, depression, education, smoking, alcohol. Association mediated by larger WC and higher urine albumin‐creatine ratio.
Brenowitz et al.^91^ USA; participants with (n = 808)/without (n = 1,366) diabetes; longitudinal, multi‐center.	In overall cohort, PN linked to incident dementia (HR 1.94), adjusted for age, sex, race, diabetes, HbA1c, hypertension, cardioVD, cerebroVD, *APOE4* status, baseline cognition, depression, cancer, cystatin C, C‐reactive protein, thyroid‐stimulating hormone), education, smoking, alcohol, sedentary lifestyle. PN in diabetes (HR 1.56) and control (HR 1.95) groups linked to incident dementia.
Ding et al.^87^ China; T1D participants (n = 70), controls (n = 48); cross‐sectional, single‐center.	CI significantly worse in T1D vs. controls. CI linked to PN (OR 8.48), age (OR 1.17), education (OR 0.30), fasting C peptide (OR 0.01), adjusted for age, sex, T1D duration, HbA1c, fasting glucose levels, BMI, serum uric acid levels, C peptide, creatinine ratio, education.
Hicks et al.^92^ USA; participants with (n = 1,072)/without (n = 2,290) diabetes; cross‐sectional, community‐based.	MCI and dementia prevalence significantly higher in participants with vs. without PN. In overall population, PN linked to MCI and dementia (OR 1.45), adjusted for age, sex, race, diabetes status, BMI, hypertension, hypercholesterolemia, cardioVD, stroke, cancer, *APOE*, education, smoking, alcohol. PN also linked to MCI and dementia in diabetes (OR 1.38) and non‐diabetes (OR 1.48) groups.
Liu et al.^93^ UK; T2D participants (n = 26,173); longitudinal, population‐based.	PN linked to all‐cause dementia (HR 1.93), AD (HR 1.95), VaD (HR 2.03), stroke (HR 1.48), ischemic stroke (HR 1.47), adjusted for age, sex, ethnicity, T2D duration, HbA1c, BMI, LDL‐c, SBP, coronary heart disease, microvascular complications (CKD, DR), drug use smoking, alcohol, exercise, diet, socioeconomic status. PN linked more strongly to dementia vs. CKD and DR.
Ming et al.^88^ Germany; T2D participants (n = 210), T1D participants (n = 51); single‐center.	PN linked to CI in full cohort (n = 261; OR 1.87) and covariate‐matched cohort (n = 178; OR 1.95), adjusted for age, sex, weight, BMI, diabetes type, diabetes duration. Predictive game features linked to PN (n = 40), CI (n = 59), or both (n = 59).
Moreira et al.^89^ Brazil; T2D participants (n = 94), controls (n = 54); cross‐sectional, single‐center.	MMSE lower in T2D vs. controls (*p* < 0.001), but not in other CI tests. No significant differences in any CI tests in T2D with vs. without PN.
Putnam et al.^94^ USA; T1D (n = 184,765) matched to T2D (n = 524,602) and controls (n = 522,768); cross‐sectional, population‐based.	Nationally representative healthcare claims database. T1D and T2D linked to cognitive disorders vs. nondiabetic controls, adjusted for microvascular/macrovascular complications. In all individuals, microvascular/macrovascular complications independently linked to cognitive disorders, adjusted for diabetes status with largest impact from PN.
Rhmari Tlemçani et al.^90^ Morocco; T2D participants (n = 100); cross‐sectional, single‐center.	PN not significantly more prevalent in T2D participants with CI. MMSE score linked to older age (*β* −0.0975), DR (*β* −1.825), dyslipidemia (*β* −3.423).
Shapiro et al.^95^ USA; T1D (n = 646) and T2D (n = 165) youths; longitudinal, population‐based.	Lowest composite and individual fluid and crystallized cognitive scores clustered with PN in T1D (along with self‐reported hypoglycemic events in the year prior to CI testing) and T2D (along with higher waist‐to‐height ratio AUC, higher SBP, higher central SBP and DBP, depression) groups.
Yen et al.^96^ Taiwan; T2D participants with (n = 79,241)/without (n = 568,577) microvascular complications; retrospective, population‐based.	National health insurance research database. PN linked to AD (HR 1.14), VaD (HR 1.14), other dementias (HR 1.10), all‐cause dementia (HR 1.12), adjusted for age, sex, obesity, co‐morbidities, medication use (antidiabetic, cardiovascular), smoking, alcohol‐related disorders.

Entries arranged alphabetically by the first author's name. *Incomplete years of study enrollment rounded to the indicated year. More detailed entries in Supplementary Table S1.

95%CI = 95% confidence interval; AD = Alzheimer's disease; *APOE4* = apolipoprotein E4; AUC = area under the curve; BMI = body mass index; cardioVD = cardiovascular disease; cerebroVD = cerebrovascular disease; CI = cognitive impairment; CKD = chronic kidney disease; DBP = diastolic blood pressure; DR = diabetic retinopathy; PN = diabetic peripheral neuropathy; HbA1c = hemoglobin A1c; HR = hazard ratio; LDL‐c = low‐density lipoprotein cholesterol; MCI = mild cognitive impairment; MMSE = Mini‐Mental State Examination; OR = odds ratio; PN = peripheral neuropathy; SBP = systolic blood pressure; T1D = type 1 diabetes; T2D = type 2 diabetes; WC = waist circumference; VaD = vascular dementia; y = year.

Population‐based studies echo these findings; in a US cross‐sectional, community‐based study, PN defined by monofilament testing was linked to MCI or dementia, by neurocognitive assessments, in participants both with and without diabetes.^92^ In a nationally representative US healthcare claims database of privately insured individuals, researchers found that microvascular complications—particularly PN—increased the odds of cognitive disorders, regardless of diabetes status.^94^ In support of this concept, in UK Biobank T2D participants, PN was linked to all‐cause dementia, AD, and VaD to a greater extent than retinopathy and chronic kidney disease.^93^ In participants with newly diagnosed T2D from the Taiwanese National Health Insurance Research Database, PN increased the odds for all‐cause dementia, AD, VaD, and other dementias in highly adjusted analyses.^96^, ^97^ Very concerningly, PN and early CI might also co‐occur in youth; in an analysis of the population‐derived prospective SEARCH study in T1D and T2D youth, PN clustered with the poorest cognitive outcomes,^95^ presaging a risk for progression to dementia in later life. Given that PN affects up to 7% of youth with T1D, and 22 to 38% of those with T2D,^14^, ^19^ this convergence represents a serious and emerging public health concern.

Finally, systematic reviews/meta‐analyses have been conducted of the relationship of PN to CI, finding no evidence,^98^ potential but unclear evidence,^99^ or supportive evidence,^100^ but all highlighting variability in tools and/or study methodologies, limiting interpretability (see section “Simultaneous events or domino effect?” and Box 2).

BOX 2 **Limitations in studies of peripheral neuropathy (PN) and cognitive impairment (CI) and required design of future studies**

The relative evolution of PN and CI in individuals with the metabolic syndrome (MetS) and/or type 2 diabetes (T2D) and obesity remains uncertain due to various limitations in study design and assessment tools. Therefore, well‐designed studies and sensitive assessment tools are needed to accurately evaluate relative development of PN and CI in individuals with the MetS and/or T2D and obesity.

**Study population characteristics**: Most studies included older participants^91^, ^92^ or participants with long‐standing metabolic dysfunction,^101^, ^102^, ^103^, ^104^, ^105^, ^106^ who were more likely to have progressive disease and associated complications. To capture the onset of both PN and CI, which would establish their relative emergence, studies must include participants of younger age,^94^ and/or with newly diagnosed MetS and/or T2D^97^ and obesity.

**Study type**: Longitudinal studies are superior for determining the relative onset of PN and CI versus cross‐sectional studies, but have been sparse.^91^, ^93^, ^96^, ^97^, ^107^ Additionally, prospective studies^91^, ^93^, ^107^ can more accurately collect potential covariates in real‐time versus retrospective studies. Center studies, even multicenter studies, may lack generalizability versus population‐based studies.

**Study population risk factors and adjusting for confounding**: In a PN exposure and CI outcome scenario, it is essential to fully adjust analyses for potential covariates to determine causality or risk. Many studies have adjusted analyses quite extensively, variably spanning demographics (age, sex, race/ethnicity), diabetes characteristics (diabetes status, diabetes duration, age at diabetes onset, HbA1c, hypoglycemia events), baseline cognition, obesity (waist circumference, body mass index), dyslipidemia, hypertension, microvascular complications (diabetic kidney disease, retinopathy), macrovascular disease (eg, cerebrovascular disease, cardiovascular disease), medical history and comorbidities (eg, cancer history, depression, comorbidity index), medication use, laboratory variables (eg, C‐reactive protein, cystatin C), genetic status (eg, *APOE4* status), lifestyle factors (smoking, alcohol use, exercise, diet), and socioeconomic variables (eg, education level, deprivation index).^86^, ^91^, ^92^, ^93^, ^96^, ^97^ Some studies have noted lack of family history on dementia as a limitation.^96^, ^97^

Studies using brain volume or white matter hyperintensities by magnetic resonance imaging have adjusted for fewer variables in general; for example, age, sex, T2D duration, total intracranial volume, education, and retinopathy severity,^92^, ^101^, ^102^, ^105^, ^108^ although other covariates may impact outcomes; for example, potential impact of obesity on regional brain and gray matter volume, and of hypertension on white matter hyperintensities. Nevertheless, in a PN marker and CI outcome scenario, studies could adjust for fewer covariates.

**PN and CI assessment tools**: Some studies used (late) diagnoses of both PN and CI; for example, using ICD codes or self‐report of pre‐existing conditions,^93^, ^94^, ^96^, ^97^ making it challenging to ascertain relative temporal onset. Alternatively, some studies used comparatively sensitive assessments tools for (earlier) PN; for example, vibration perception threshold, nerve conduction studies, but a (later) diagnosis of CI; for example, hospitalization for dementia, prescribed dementia medication use.^91^ Yet still, some studies used tests for symptoms and signs of PN; for example, 10‐g monofilament testing,^92^ Michigan Neuropathy Screening Instrument,^106^ but functional; for example, neurocognitive assessments,^92^, ^106^ and structural surrogates; for example, brain/gray matter volume, cortical thickness by magnetic resonance imaging,^92^, ^106^ of CI. A multimodal framework is needed to assess both PN and CI that spans symptoms, signs, neuroelectrophysiological, structural, and functional tests.

**Mechanisms underlying PN and CI**: Some investigations have used too broad a definition of dementia; for example, ICD codes that included Pick's disease and dementia with Lewy bodies.^96^ In the context of PN and CI pathophysiology stemming from shared metabolic risk factors, it is critical to accurately define PN and CI as metabolically acquired.

**Study size**: Small studies can bias findings and lack generalizability to larger cohorts. Although most studies of PN and CI were large, those that leveraged neuroelectrophysiological and structural (magnetic resonance imaging) methods were generally, albeit understandably, small.

**Future directions**: Large, population‐based, prospective, and longitudinal studies are needed to accurately assess the aggregation and relative evolution of PN and CI using participants with early metabolic dysfunction (ie, newly diagnosed T2D, obesity, MetS), and a multimodal assessment framework of PN and CI. The prospective design will facilitate comprehensive collection of covariates in real‐time to fully adjust analysis for confounding.

### PN Correlates to Brain Structural Changes in Diabetes

In addition to PN correlations to CI or dementia, studies have also examined the link to brain structural and functional changes (Table 2; reviewed in Wang et al^109^), although none were noted in earlier studies.^107^, ^108^ For example, a Turkish study found that deep white matter lesions were more frequent in T2D participants with versus without PN,^101^ and brain structural changes in the right orbitofrontal cortex already occur in T2D patients with subclinical PN.^110^ Independent of diabetes, in a large US community‐based cohort, participants with versus without PN showed both CI and lower total and lobar brain volumes, but no alterations in white matter hyperintensities, microhemorrhages, or infarcts.^92^

**TABLE 2:** Summary of Studies of Peripheral Neuropathy or Diabetic Peripheral Neuropathy (PN) to Brain Structural Changes in Patients with or Without Diabetes

Study location, participants, design	Study tools	Main study findings
Ferik et al.^101^ Turkey; T2D participants (n = 66); cross‐sectional, single‐center.	PN symptoms, signs, NCS by Toronto Consensus criteria; neuropathic symptoms by LANSS, DN4, NDS, NSS; CI by Mini‐Mental State Examination; brain imaging by MRI.	Deep white matter lesions more common in participants with vs. without PN, adjusted for age, T2D duration. In PN participants, deep white matter lesions linked to older age and longer T2D duration.
Hansen et al.^105^ Denmark; T1D participants (n = 48), controls (n = 28); cross‐sectional, single‐center.	PN by sural, peroneal, tibial nerves EMG vs. age‐matched controls to generate a clinical neurophysiological composite z‐score; painful PN by DN4; total and regional GM volume, cortical thickness by MRI.	Lower total GM volume in T1D participants with PN, painful PN, or retinopathy vs. controls (*p* < 0.05 for all), adjusted for age, total intracranial volume. Total GM volume linked to PN, T1D duration, age, and parietal N‐acetylaspartate/creatine but not glycated hemoglobin A1c. GM volume loss independently linked to older age, more severe PN, and lower parietal N‐acetylaspartate/creatine.
Hicks et al.^92^ USA; participant subset with available MRI (n = 1,095); cross‐sectional, community‐based.	Brain imaging by MRI.	Total (*p* < 0.05) and lobar (*p* < 0.05) brain volumes lower in participants with vs. without peripheral neuropathy, adjusted for age, but no differences in white matter hyperintensity, lobar microhemorrhages, subcortical microhemorrhages, cortical and lacunar infarcts.
Reynolds et al.^106^ Gila River Community, USA; T2D participants (n = 51); cross‐sectional, single‐center.	PN by Michigan Neuropathy Screening Instrument; CI by NIHTB‐CB; brain imaging by structural/functional MRI.	T2D duration, but no other metabolic risk factors, linked to lower cortical thickness and GM volume and higher white matter hyperintensity volume. Participants had similar NIHTB‐CB composite score vs. normative data.
Selvarajah et al.^104^ United Kingdom; T1D and T2D participants without PN (n = 57), with painful PN (n = 77), with painless PN (n = 77), age‐ and sex‐matched controls (n = 66); cross‐sectional, observational, single‐center.	PN by NCS; painful PN by the International Association for the Study of Pain definition of neuropathic pain and DN4; sensory phenotyping by QST to classify painful PN as irritable (IR) vs. nonirritable (NIR) nociceptor phenotypes.	Primary somatosensory and motor cortical less thick in painful and painless PN vs. controls (*p* = 0.02) and participants without PN (*p* = 0.01). Somatomotor cortical thickness linked to PN severity. Ventrobasal thalamic nuclei volume lower in both painless and painful PN. Lower primary somatosensory cortical, posterior cingulate cortical, and thalamic volume in painful PN participants with NIR vs. IR nociceptor phenotypes. Analyses adjusted for age and sex.
Zhao et al.^110^ China; T2D participants without PN (n = 20), subclinical PN (n = 12), with PN (n = 23); cross‐sectional, single‐center.	PN by NSS, NDS, DN4, NCS, QST, classified participants as without PN, subclinical PN, and with PN; brain morphology and function by MRI.	Orbitofrontal to bilateral postcentral and middle temporal cortex functional connectivity increased in T2D participants with subclinical vs. without PN. Orbitofrontal GM volume and functional connectivity transiently increased in T2D participants with subclinical vs. with PN. Orbitofrontal cortex GM volume negatively linked to NSS, NDS, and DN4 and positively linked to bilateral peroneal amplitude and right sensory sural nerve conduction velocities. Orbitofrontal to postcentral cortex functional connectivity positively linked to cold thermal perception threshold and negatively linked to NSS.

Entries arranged alphabetically by the first author's name. *Incomplete years of study enrollment rounded to the indicated year. More detailed entries in Supplementary Table S1.

CI = cognitive impairment; DN4 = Douleur Neuropathique en 4 Questions; EMG = electromyography; GM = grey matter; LANSS = Leeds Assessment of Neuropathic Symptoms and Signs; NDS = Neuropathy Disability Score; NIHTB‐CB = NIH Toolbox Cognition Battery; NSS = Neuropathy Symptom Score; PN = diabetic peripheral neuropathy; QST = quantitative sensory testing; T1D = type 1 diabetes; T2D = type 2 diabetes.

Magnetic resonance imaging investigations have also been conducted on participants with T1D and PN, both painless and painful, although most focused on gray matter loss in the corresponding somatosensory regions, which were more pronounced with more severe neuropathy.^102^, ^103^, ^104^ It is possible that structural changes to somatosensory brain regions in PN patients might indicate “atrophy” resulting from deficient peripheral stimulation and consequent plasticity or alternatively from neurodegeneration more generally. Broader brain changes also occur in T1D participants with PN; for example, to primary motor cortex,^104^, ^105^ posterior cingulate cortex,^104^ frontal cortex,^105^ occipital cortex,^105^ cingulate gyrus,^102^ and thalamus.^104^, ^105^

Overall, although many magnetic resonance imaging studies emphasized somatosensory regions, some indicate potential correlation of PN with injury to brain areas or networks controlling or linked to cognition,^92^, ^101^, ^102^ although further investigation is needed.

## MetS‐Induced PN and CI: Simultaneous Events or Domino Effect?

Mounting evidence indicates that PN and CI/dementia aggregate in MetS patients, yet their relative relationship remains uncertain due to design limitations in studies conducted to date and tools used. Does PN develop simultaneously with or ahead of early CI (Box 2)? Most studies have been cross‐sectional, precluding evaluation of the relative temporal evolution of PN to CI. Furthermore, assessment tools for PN and CI have differed in their sensitivity for early detection; for example, electrophysiological tests for early PN versus end‐stage diagnoses for dementia,^91^ or end‐stage diagnoses for both,^93^, ^94^, ^96^ preventing determination of their relative onset and development. Furthermore, most studies to date were of older participants^91^, ^92^ or participants with long‐standing diabetes,^101^, ^104^, ^105^, ^106^ more liable to have progressive associated complications; thus, the window for detecting PN and early CI onset would have passed. Multimodal longitudinal assessments of various PN and CI measures using diverse tests; for example, symptoms, signs, electrophysiological, structural, functional, and so on in younger participants^94^ with newly diagnosed diabetes^96^ will be required to elucidate the relative onset of PN and early CI with MetS progression.

It will also be essential to delineate the true relationship between PN and CI/dementia. It is feasible to treat PN as the exposure and dementia as the outcome in an epidemiological sense; many studies have substantially adjusted for confounding in this exposure–outcome scenario, but few have comprehensively addressed all potential confounding. It is also possible to argue that end‐stage damage occurs to several organs with longer MetS duration, and that the relationship of PN to dementia is not unique, but also occurs with, for instance, retinopathy and nephropathy. We propose a potentially special PN–CI relationship due to shared risk factors and pathophysiology, and particularly due to vulnerabilities that arise from the unique attributes and needs of neurons; for example, intense energy demands rendering them susceptible to metabolic dysfunction (see section “Impaired mitochondrial dynamics in MetS PN and CI”).

The largest studies have noted the greatest effect sizes for neuropathy over retinopathy and nephropathy to CI/dementia,^93^, ^94^, ^95^, ^97^ although this did not uphold in some smaller studies.^90^, ^105^, ^107^ If both arise from shared risk factors, PN may alternatively be viewed as an early clinical biomarker for CI and of brain health status more broadly. In this scenario, analyses may not need to adjust as extensively for confounding, but rather, identify mediating factors. Future investigation in diverse settings will be needed to account for the possible structures of the relationship between PN and CI, and whether it is unique versus other end‐stage complications. Furthermore, this review focused on shared metabolic risk in PN and CI, although they might feasibly share genetic risk or might be causally related through gene variants,^111^ which could be an area of future investigation.

**FIGURE 3:**
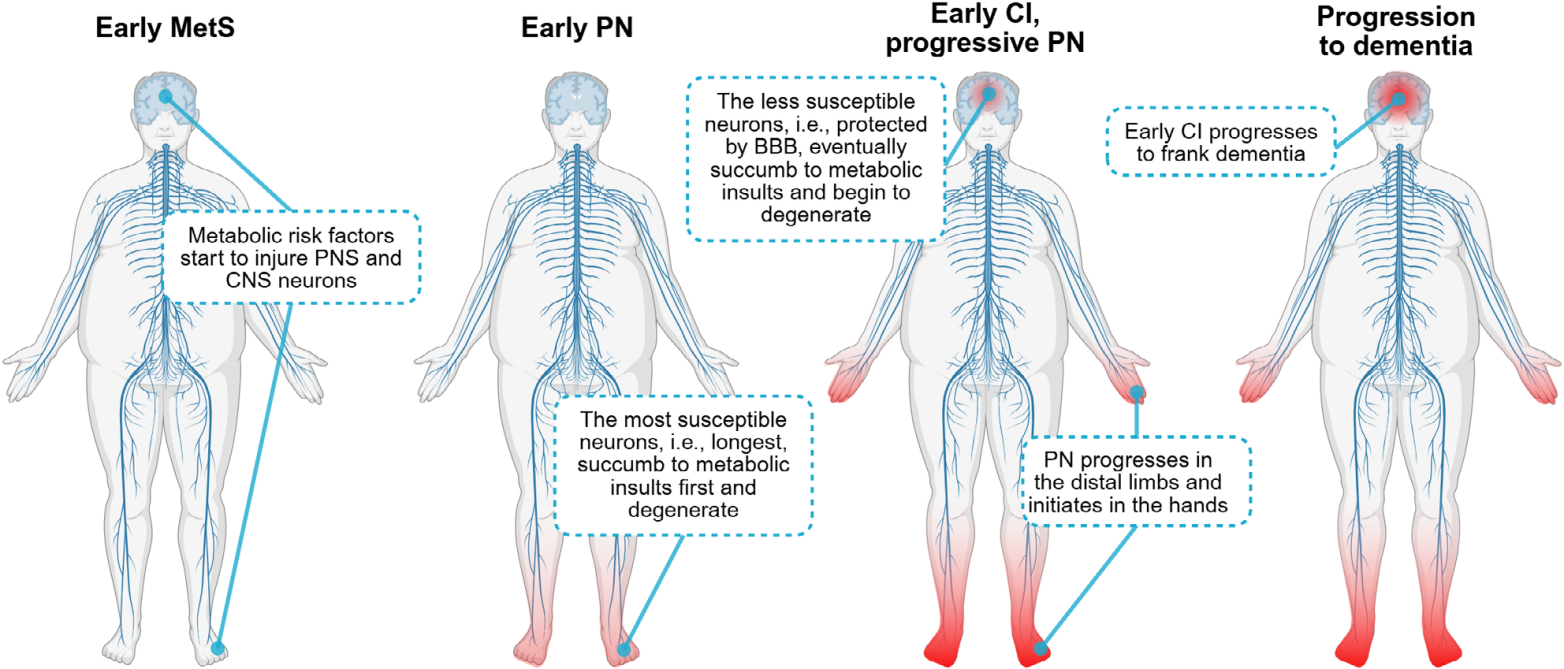
The proposed progression of the “stocking–glove–hat” pattern in patients with the metabolic syndrome. BBB = blood–brain barrier; CI = cognitive impairment; CNS = central nervous system; MetS = metabolic syndrome; PN = peripheral neuropathy; PNS = peripheral nervous system. Created in BioRender.com. [Color figure can be viewed at www.annalsofneurology.org]

Importantly, our “stocking–glove–hat” formulation does not hypothesize a single neurodegenerative continuum nor that PNS neurodegeneration directly impacts CNS processes. Rather, we propose that shared MetS risk factors injure neurons through shared pathophysiology, be they in the PNS or CNS. In this context, a person with the MetS is at risk of developing PN, as well as being at risk of developing CI. Thus, in the “stocking–glove–hat” framework, a person with the MetS and PN (ie, with stocking‐glove) has been exposed to the risk factors that injure neurons and is at higher risk of developing CI (ie, the hat; Fig 3).

Although definitive evidence is lacking, we propose that PN develops ahead of CI onset. First, the distal‐to‐proximal symptomology in PN and underlying peripheral nerve injury arise from their long aspect ratio renders the most distal parts susceptible to MetS‐induced breakdown in the cellular processes that normally sustain neurons.^43^ In contrast, CNS neurons have a more moderate aspect ratio (Fig 2A). Second, peripheral nerves and DRG reside behind a blood–nerve barrier,^49^ and are more vulnerable to systemic insults; for example, hyperglycemia, than brain neurons, defended by a more stringent blood–brain barrier (Fig 2B),^112^ with consequent PN onset before CI. A preclinical study noted early defects in mitochondria and lipids in peripheral nerve in the T2D disease course, with ensuing CNS manifestation,^79^ suggesting PN might occur ahead of CI.

## What are the Implications of the Interrelationship of MetS‐Induced PN and CI?

**FIGURE 4:**
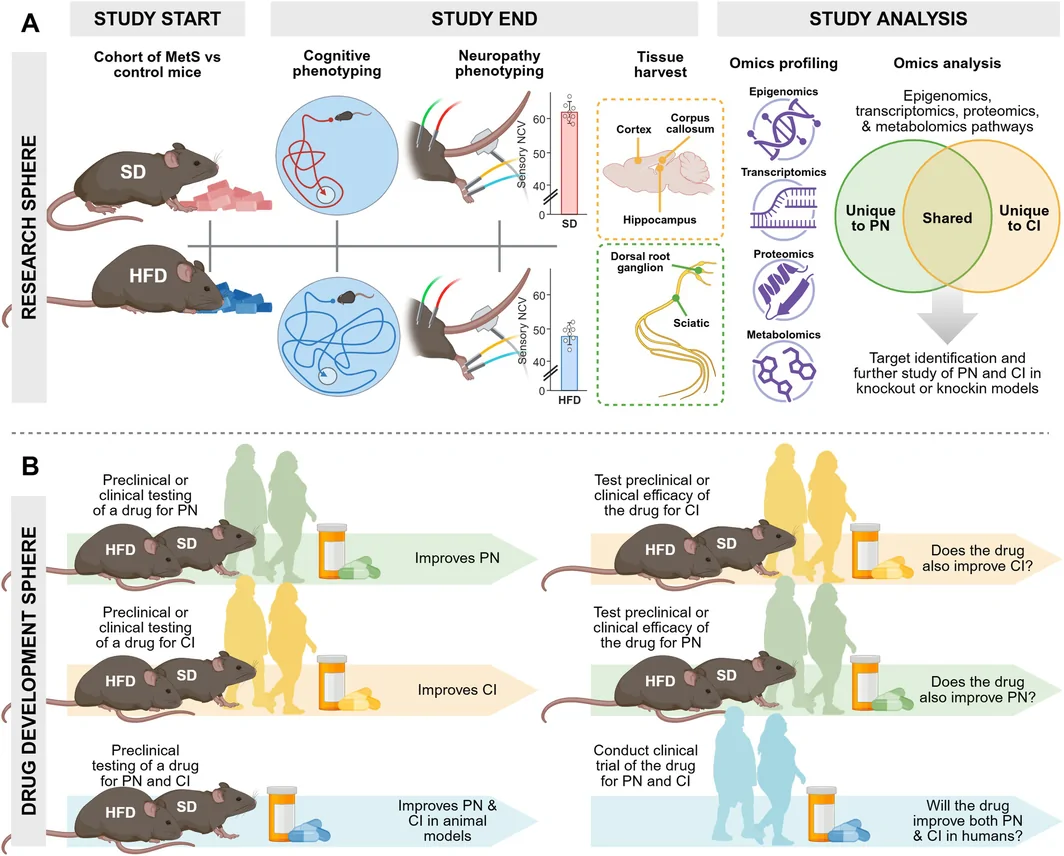
Research and drug development implications of the interrelationship of metabolic syndrome‐induced peripheral neuropathy (PN) and cognitive impairment (CI). (A) Research implications of the interconnectedness of PN and CI in cohorts of rodents with metabolic syndrome versus controls. Shown for mice with high‐fat diet‐induced obesity and prediabetes, but experiments could use various alternative models; for example, high‐fat diet‐streptozotocin type 2 diabetes mice, *db*/*db* and *ob*/*ob* type 2 diabetes with deficient leptin signaling. Metabolic syndrome mice develop CI (shown using the Morris water maze although various tests can be employed) and PN (shown using sensory nerve conduction velocity although various tests can be employed). Agnostic, system‐wide analyses of tissue can identify shared and unique molecular pathways in PN and CI, pinpointing targets for further investigation. (B) Top, potential development trajectory of a drug found effective for PN; middle, potential development trajectory of a drug found effective for CI; bottom panel, new paradigm for the development of a drug effective against both PN and CI. CI = cognitive impairment; HFD = high‐fat diet; MetS = metabolic syndrome; NCV = nerve conduction velocity; PN = peripheral neuropathy; SD = standard diet. Created in BioRender.com. [Color figure can be viewed at www.annalsofneurology.org]

If a connection between PN and CI is substantiated, it would have important consequences for research, therapeutic development, and clinical care. In the research sphere, PNS and CNS preclinical models could facilitate tandem investigations into the respective pathophysiology of MetS‐induced PN and CI (Fig 4A). We urge PNS investigators to consider expanding their research into the CNS and vice versa. For instance, we recently concluded parallel preclinical studies of the effects of relatively long‐term ketogenic diet on both MetS‐induced PN^45^ and CI.^47^ Parallel preclinical investigation of both PN and CI might identify novel shared underlying mechanisms while also identifying distinct pathways arising from the unique PNS and CNS microenvironments; for example, the blood–brain versus blood–peripheral nerve barrier,^49^, ^112^ differing extents in immune privilege or neuroimmune communication,^113^, ^114^ support from distinct glia^43^, ^70^ and so on. Dually exploring PN and CI can maximize discovery from the same preclinical cohorts and pinpoint new actionable therapeutic targets.

In the therapeutic development sphere, due to shared risk factors and pathophysiology, it is feasible that avenues effective for PN could potentially also work for MetS‐related CI and/or dementia, and vice versa (Fig 4B).^115^ The current body of clinical studies showcase this as a potentially viable concept. Lifestyle habits or interventions, such as exercise,^116^, ^117^ dietary regimens,^118^, ^119^ and bariatric weight‐loss surgery,^120^, ^121^ might exert salutary effects to improve, stabilize, or prevent both early PN^116^, ^118^, ^120^ and CI.^117^, ^119^, ^121^ Emerging results from investigations for diabetes‐induced CI, for instance of glucagon‐like peptide 1 receptor agonists,^122^ should also prompt analysis for PN in the PNS, which also expresses glucagon‐like peptide 1 receptors,^123^ but has been far less explored,^124^ and vice versa. Even more systematically, therapeutic discovery could launch simultaneously, testing early candidates in preclinical MetS models for both PN and CI,^115^ accelerating the drug development pipeline. Additionally, and optimally, it will be important to determine whether preventing PN and managing associated risk factors also mitigate CI/dementia.

**FIGURE 5:**
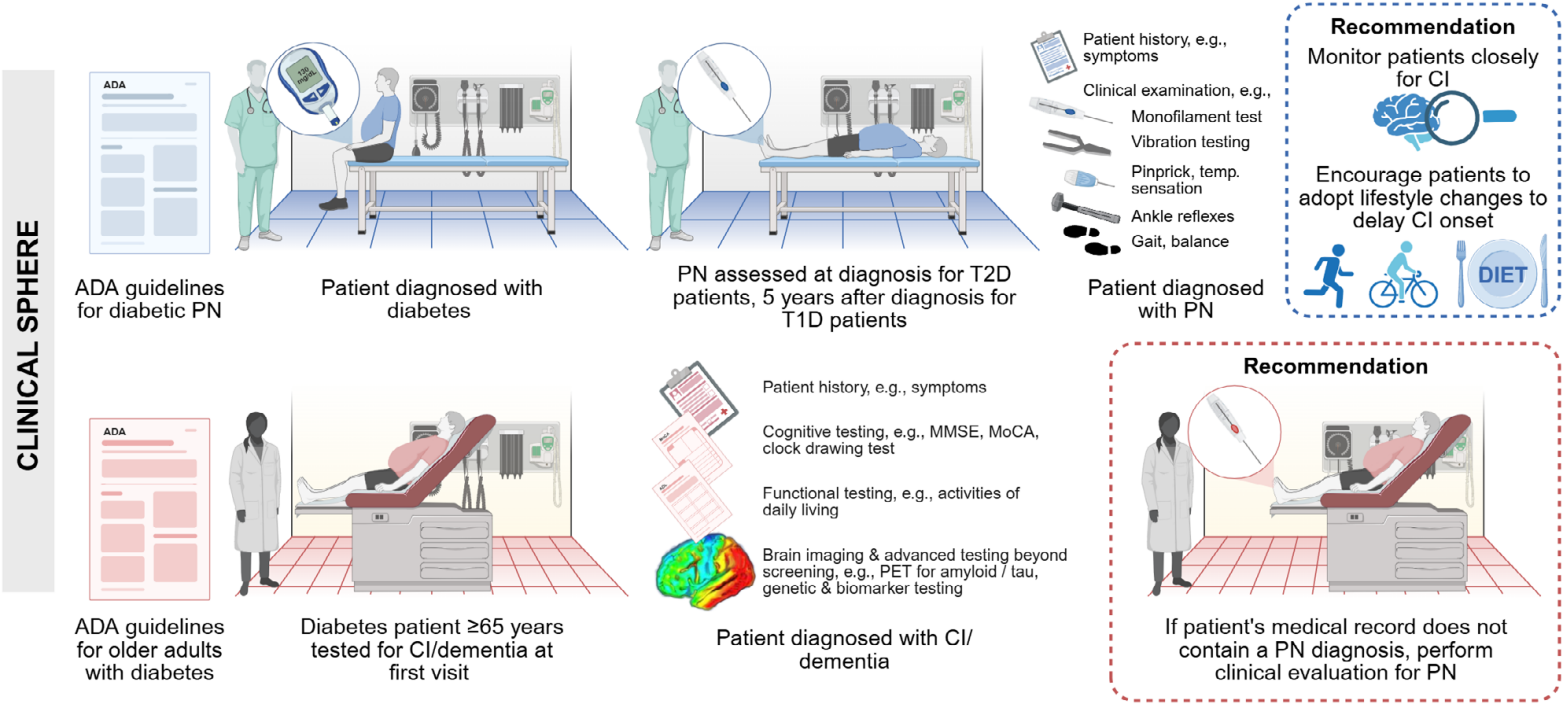
Clinical implications of the interrelationship of metabolic syndrome‐induced peripheral neuropathy and cognitive impairment. Clinical implications of the interconnectedness of peripheral neuropathy and cognitive impairment for patient care. Upper: potential care trajectory of a diabetes patient diagnosed with peripheral neuropathy; lower: potential care trajectory of an older diabetes patient diagnosed with cognitive impairment. ADA = American Diabetes Association; CI = cognitive impairment; MMSE = Mini‐Mental State Examination; MoCA = Montreal Cognitive Assessment; PET = positron emission tomography; PN = peripheral neuropathy; T1D = type 1 diabetes; T2D = type 2 diabetes; Temp = Temperature. Created in BioRender.com. [Color figure can be viewed at www.annalsofneurology.org]

In the clinical care sphere, although seen as the domains of distinct neurological subspecialties affecting the PNS or CNS, MetS‐induced PN and CI might be increasingly viewed as connected conditions. A PN and CI/dementia interrelationship in patients with MetS, if further substantiated, would have important ramifications for screening (Fig 5). The American Diabetes Association guidelines and standards of care for older adults with diabetes recommend early screening for mild CI or dementia in adults aged ≥65 years at the initial visit, annually thereafter, or as appropriate.^125^ In parallel, the American Diabetes Association guidelines and standards of care for PN recommend screening at T2D diagnosis and 5 years after T1D diagnosis, and annually thereafter.^31^, ^126^ However, in light of the relationship between PN and CI/dementia, we believe the guidelines could be amended to screen all PN patients, including those aged <65 years, for CI on PN diagnosis and annually thereafter.

Youths with PN are an especially vulnerable population that merit particular care and attention to mitigate future risk of dementia as much as possible. Conversely, an older person with diabetes found to have mild CI or dementia should automatically prompt assessment for PN, if their medical record does not already contain such a diagnosis. The tandem occurrence of PN and CI has important implications for early detection through biomarkers, which could assess the central and peripheral compartments jointly, such as neurofilament light chain common to both, or separately, such as PNS‐specific peripherin.^127^

Finally, besides MetS‐acquired PN and dementia, it is also feasible to consider their interconnectedness in other contexts. For example, emerging evidence suggests T2D is associated with hearing loss,^128^ which is also an early sign of dementia.^2^ Based on our central premise, in persons with the MetS, we would therefore anticipate aggregation of hearing loss with PN, especially if the underlying pathophysiology of hearing loss involves vascular and/or neural dysfunction related to metabolic risk factors.^128^ Early evidence suggests that diabetic PN might be linked to hearing loss in T2D patients,^129^, ^130^ but further analyses are warranted.

## Conclusions

A growing body of evidence indicates that PN and CI/dementia, which have overlapping risk factors and pathophysiology, both occur in diabetes and MetS patients, reflecting tandem neurodegeneration in the PNS and CNS. Prospective, longitudinal studies using sensitive and repeated measures of PN and early CI are needed to more definitively determine their relative onset. Nevertheless, we propose that the “stocking–glove” model of PN can be extended to a “stocking–glove–hat” model, which also encompasses CNS nerve damage and CI/dementia in individuals with diabetes and the MetS. The “stocking–glove” depiction of PN airs a simplicity as a phenotypic descriptor of sensory loss, a teaching that generations of medical trainees have successfully retained. We suggest adding the “hat” as a prompt to draw attention to the CNS complications of diabetes and the MetS, and to encourage early screening and diagnosis of CI and dementia. Furthermore, if examinations into PN and CI/dementia continue to support that they aggregate in individuals with diabetes and the MetS, it could advocate for a realignment in our approach to research, clinical care, and therapeutic development.

## Author Contributions

M.G.S. and E.L.F. contributed to the conception and design of the manuscript; M.G.S. contributed to the interpretation of studies included in the manuscript; all authors contributed to drafting the text and preparing the figures.

## Potential Conflicts of Interest

ELF is an Associate Editor at the *Annals of Neurology*. All other authors nothing to report.

## Supporting information


**Table S1.** Summary of studies of cognitive impairment (CI) and peripheral neuropathy (PN).

## Data Availability

Not applicable.
