# Supplementary material for: Diabetes and the Metabolic Syndrome as Drivers of Neurodegeneration: Convergent Mechanisms Linking Peripheral Neuropathy and Dementia
Source: Ann Neurol. 2026 Jul 7;100(3):458–76. doi: 10.1002/ana.78289 (PMC13495836; doi:10.1002/ana.78289)
Supplement: Supplementary file 1 [file ANA-100-458-s001.pdf]

SUPPLEMENTARY INFORMATION FOR

Metabolic syndrome and neurodegeneration: A unified view of peripheral neuropathy and dementia

Masha G. Savelieff,[1,*] David L. Bennett,[2] Troels S. Jensen,[3,4] Eva L. Feldman[5,6]

[1]Department of Biomedical Sciences, University of North Dakota, Grand Forks, ND 58202, USA

[2]Nuffield Department of Clinical Neuroscience, University of Oxford, Oxford OX3 9DU, UK

[3]Department of Neurology, Aarhus University Hospital, Aarhus, Denmark

[4]Danish Pain Research Center, Aarhus University, Aarhus, Denmark

[5]Department of Neurology, University of Michigan, Ann Arbor, MI 48109, USA

[6]NeuroNetwork for Emerging Therapies, University of Michigan, Ann Arbor, MI 48109, USA

*Correspondence to: Masha G. Savelieff, PhD

Email: masha.savelieff@ndus.edu

Supplementary Table S1. Summary of studies of cognitive impairment (CI) and peripheral neuropathy (PN)

Entries arranged alphabetically by the first author's name.

*Incomplete years of study enrollment rounded to the indicated year.

**Limitations and strengths as cited by papers.

Study	Country & region	Time*	Study participants	Study design	Study tools	Study findings	Study limitations/strengths**
**Barzilay et al.[1]**	USA	Not stated	5,047 T2D participants	Cross-sectional analysis of prospective, multi-center cohort	Glycemia Reduction Approaches in Diabetes study (GRADE). Excluded participants with any major cardioVD event in the year prior to recruitment and/or a history of New York Heart Association heart failure stages 3 or 4. DPN by MNSI exam and questionnaire; CI by SEVLT, DSST, and animal and letter fluency. CAN, metabolic, diabetic complications tests.	Mean age (SD) 58.6 (9.3) y with and 56.2 (10.0) without DPN. Mean T2D duration (SD) 4.2 (2.7) y with and 4.0 (2.8) y without DPN. DPN (continuous or dichotomous), but not CAN, linked to various CI outcomes, adjusted for age, sex, race, WC, diabetes duration, hypertension, hyperlipidemia, smoking, alcohol, stroke history, depression, education. Larger WC and higher urine ACR mediated this association.	***Limitations***: Cross-sectional, CAN measures based only on time domain variables. ***Strengths***: Large, diverse, well characterized cohort using known DPN, CAN, CI tests. DPN and CAN modeled as continuous and categorical variables.
**Brenowitz et al.[2]**	USA	1997-1998 + 16 y follow-up	2,174 participants	Longitudinal, multi-center, cohort	Health, Aging, and Body Composition study (Health ABC). Sensory PN by VPT and 10g monofilament, motor PN by CMAP and NCV. Incident dementia by 1 or more of recorded hospitalization for dementia (primary or secondary diagnosis), self-report (hospital admissions, dementia medications, annual visits with modified MMSE), documented prescribed dementia medication use. Medical history, metabolic/laboratory tests.	Mean age 76.8 (2.9) y with and 76.4 (2.8) y without PN impairments. In overall cohort, sensory PN by VPT linked to incident dementia (HR 1.94, 95%CI 1.31-2.86), adjusted for age, sex, race, education, hypertension, diabetes, cerebroVD, cardioVD, smoking, alcohol, sedentary lifestyle, depressive symptoms, cancer history, cystatin C, CRP, HbA1c, TSH, *APOE4* status, baseline cognition. Sensory PN by monofilament in diabetes group (HR 1.56, 95%CI 1.02-2.37) and VPT in control group (no diabetes, normal B12, no *APOE4*, HR 1.95, 95%CI 1.07-3.55) linked to incident dementia, adjusted for age, sex, race, education, hypertension, diabetes (for non-diabetes models), cerebroCV, cardioCV, smoking, alcohol, sedentary lifestyle, depressive symptoms. Graded increase in HR for incident dementia with more sensory and motor impairment. Largest effect from sensory impairment.	***Limitations***: Nerve function measured only in one lower limb extremity at single time point. Lacked data on potential drug-induced PN (but adjusted for cancer history), other measures of vitamin B12-related deficiencies, and location, mechanisms, and duration of nerve injury. Dementia by established criteria but not by clinical diagnosis, may have been misclassified. Some missing data. Uncertain generalizability. ***Strengths***: Large, prospective, long follow-up, objective sensory and motor PN measures, established all-cause dementia definition, participants with/without diabetes, adjusted analyses.

Study	Country & region	Time*	Study participants	Study design	Study tools	Study findings	Study limitations/strengths**
**Chang et al.[3]**	Taiwan	2001-2013	173,118 newly diagnosed T2D participants	Longitudinal, population-based cohort	National Health Insurance Research Database. Excluded participants with dementia diagnosis or that developed vascular diseases within 1 y prior to T2D diagnosis date. Diabetes-related microvascular (DPN, retinopathy, nephropathy) and macrovascular (CHD, PAD, stroke) diseases and incident dementia (VaD, AD, other dementia types) identified by ICD-9 Clinical Modification codes and/or procedure codes in inpatient, outpatient, or emergency department records. Comorbidities from medication use.	Baseline mean age 67 (9.0) y and mean T2D duration 2.78 (3.0) y in overall population; 1,864,279 person-years of follow-up. DPN linked to all-cause dementia (HR 2.06, 95%CI 1.99-2.14), but also presence of one vascular disease (HR 1.99, 95%CI 1.92-2.07), presence of ≥2 vascular diseases (HR 2.04, 95%CI 1.98-2.13), stroke only (HR 3.56, 95%CI 3.44-3.70), nephropathy, retinopathy, cardioVD, or PAD, adjusted for age, sex, diabetes duration, comorbidities (hyperlipidemia, hypertension, hypoglycemia, arrhythmia, depression), medication use (antidiabetic, antihypertensive, lipid-lowering, antidepressants, antiplatelet), number of metabolic and hypoglycemic events during follow-up. DPN (and other conditions) linked to AD and VaD individually.	***Limitations***: Observational study, dementia onset uncertain. Dementia diagnosis by codes, possible misclassifications. Potentially underestimated dementia incidence due to mild cases that did not seek care. Lacked data on HbA1c, family dementia history, smoking, alcohol, education, SES, alternative PN causes. Uncertain generalizability. ***Strengths***: Few participants lost to follow-up. Used conservative algorithm to identify diabetic vascular diseases, eliminated confounding from stroke, and considered duration between diabetes and vascular disease diagnosis to reduce immortal time bias. Consistent original and sensitivity analyses in matched cohorts.
**de Bresser et al.[4]**	Netherlands	Not stated	68 older T2D participants; MRI scans available for 55 T2D participants	Longitudinal, population-based cohort	Utrecht Diabetic Encephalopathy Study. Excluded participants with psychiatric or neurological disorder (unrelated to T2D) that could influence CI, alcohol or substance abuse, or dementia. Baseline DPN by modified Toronto Clinical Neuropathy Scoring System. CI at baseline and follow-up by neuropsychological exams in 11 verbal and nonverbal tasks in 5 cognitive domains. Brain volume by MRI. Baseline retinopathy, nephropathy, metabolic variables.	Baseline mean age 65.6 (5.6) y and T2D duration 9.1 (6.3) y in overall cohort; 4 y follow-up; baseline DPN, retinopathy, and nephropathy not linked to baseline composite CI. Baseline nephropathy, but not DPN or retinopathy, linked to lower composite CI, but not individual domains, at follow-up.	***Limitations***: Relatively small study, possible selection bias due to attrition but no demographic differences in participants with those lost to follow-up. Lacked MRI scans for all participants. Relied on p<0.01. Participants recruited from general population with relatively mild microvascular complications; uncertain generalizability. ***Strengths***: Prospective, detailed CI and microvascular assessment, precise brain volume measures.
**Ding et al.[5]**	China	2013-2014	70 T1D participants, 48 controls	Cross-sectional, single-center, case-control cohort	Excluded participants with a disease that may cause CI and with educational level ≤ 6-year. DPN by NCV; CI by MMSE, MoCA. Retinopathy. Clinical variables.	Mean age (SD) 32.2 (9.6) y T2D and 31.6 (7.2) y in controls; mean T2D duration (SD) 9.0 (7.0) y. MMSE (p=0.005) and MoCA (p=0.017) scores poorer in T1D vs. controls. CI by MoCA linked to age (OR 1.17, 95%CI 1.02-1.35, p=0.025), NCV (OR 8.48, 95%CI 1.96-81.34, p=0.046), education (OR 0.30, 95%CI 0.03-2.80, p=0.019), fasting C peptide (OR 0.01, 95%CI 0.00-0.51, p=0.023), adjusted for age, sex, T1D duration, HbA1c, fasting glucose levels, BMI, serum uric acid levels, C peptide, creatinine ratio, education.	***Limitations***: Relatively small study; cross-sectional, cannot assess causality. HbA1c assayed once, so relationship between blood glucose levels and CI not elucidated. Similarly, few diabetic ketoacidosis or severe hypoglycemia events, not considered in their relation to CI. ***Strengths***: None specifically stated.
**Elsharkawy et al.[6]**	Egypt	2019-2020	45 T2D participants with DPN, 45 age-/sex-matched controls	Cross-sectional, single-center, case-control cohort	Excluded participants for various criteria, including heart failure, PVD, retinopathy, drug or alcohol abuse, or any disease other than T2D linked to neuropathy or might interfere with assessments. DPN by NSS and severity by Dyck grading; CI by MMSE, P300. Clinical variables, serum neuron-specific enolase levels.	Age 26-60 y in T2D and 27-59 y in controls; median T2D duration (IQR) 11 (8-16) y. Participants had painless (n=21) and painful (n=24) DPN. MMSE and P300 scores poorer in T2D vs. controls (p<0.001 for both). P300 scores, but not MMSE, differed significantly in painful vs. painless T2D DPN (p=0.03). P300 scores correlated significantly with HbA1c and serum neuron-specific enolase levels.	***Limitations***: None specifically stated. ***Strengths***: None specifically stated.

Study	Country & region	Time*	Study participants	Study design	Study tools	Study findings	Study limitations/strengths**
**Ferik et al.[7]**	Turkey	Not stated	66 T2D participants	Cross-sectional, single-center cohort	Excluded participants with prior PN-causing disorders other than T2D, cerebroVD, cardioVD, hypertension, or smoking, alcohol, or substance abuse. DPN by symptoms, signs, NCS by Toronto Consensus criteria; neuropathic symptoms by LANSS, DN4, NDS, NSS; CI by MMSE; brain imaging by MRI. Carotid Doppler sonography, metabolic/laboratory tests.	Mean age 54.5 (7.6) y and mean T2D duration 114.5 (75) mo in overall cohort. Deep WM lesions (DWMLs) more common in participants with vs. without DPN, adjusted for age, T2D duration. In DPN participants, DWMLs linked to older age and longer T2D duration. No significant differences in carotid atherosclerosis in participants with vs. without DPN or DWMLs.	***Limitations***: None specifically stated. ***Strengths***: None specifically stated.
**Hansen et al.[8]**	Denmark	2014-2017	48 T1D participants (46 with MRI scans), 28 controls	Cross-sectional, single-center case-control cohort	Excluded participants with T2D, other neurological disorders than DPN, nephropathy. DPN by sural, peroneal, tibial nerves EMG relative to age-matched controls to generate a clinical neurophysiological composite score based on z-scores. Painful DPN by DN4. Total and regional GM volume and cortical thickness by MRI. Retinopathy, clinical, metabolic variables.	Mean age (SD) 50.0 (8.5) y in T1D and 49.9 (11.9) y in controls; mean T1D duration (SD) 32.2 (9.5) y in T1D cohort. T1D participants with DPN, painful DPN, or retinopathy had reduced total GM volume vs. controls ($p<0.05$ for all), adjusted for age, total intracranial volume. Total GM volume linked to DPN, T1D duration, age, and parietal N-acetylaspartate/creatine (NAA/cre) ratio but not HbA1c. Older age, more severe DPN, and lower parietal NAA/cre ratio independently linked to GM volume loss.	***Limitations***: Relatively homogeneous cohort; cross-sectional, cannot assess causality. Focused on whole brain morphology and lacked regional atrophy and cortical thinning. Imbalance in sex, but analyses adjusted for total intercranial volume. ***Strengths***: Relatively large cohort.
**Hicks et al.[9]**	USA	2016-2017	3,362 participants	Cross-sectional, community-based	Atherosclerosis Risk in Communities Neurocognitive Study (ARIC-NCS). PN by 10g monofilament. Participants classified as normal, MCI, or dementia by an adjudication committee based on comprehensive battery of neurocognitive assessments. Brain imaging by MRI. Sociodemographic and clinical variables.	Mean age (SD) 80.5 (5.0) y with and 78.6 (4.3) y without PN. MCI (21.9% vs. 16.7%, $p<0.05$) and dementia (7.8% vs. 3.9%, $p<0.05$) prevalence higher in participants with vs. without PN. PN linked to MCI or dementia in overall study population (OR 1.45, 95%CI 1.23-1.73), adjusted for age, sex, race, diabetes status, BMI, cardioVD, stroke, hypertension, hypercholesterolemia, cancer, *APOE*, education, smoking, alcohol. Similar by diabetes (OR 1.38, 95%CI 1.03-1.87) and no diabetes (OR 1.48, 95%CI 1.20-1.83) status. Age-adjusted total ($p<0.05$) and lobar ($p<0.05$) brain volumes lower in participants with vs. without PN (in subset with available MRI, n=1,095), but no differences in WMH, lobar microhemorrhages, subcortical microhemorrhages, cortical and lacunar infarcts.	***Limitations***: Cross-sectional, PN by monofilament, not NCS gold standard, monofilament may not be as accurate in participants with vs. without CI. Participants with advanced dementia may not have attended the relevant study visit, leading to selection bias. PN and brain MRI non-concurrent, collected ca. 3-5 y apart. ***Strengths***: Large cohort size, CI assessed robustly by a formal adjudication committee, PN assessed by standardized monofilament testing.
**Karunathilaka et al.[10]**	Various countries	Included studies 1984-2022	Diabetic participants	Systematic review and meta-analysis, 13 studies	Included studies of diabetic participants (population) with diabetes-related lower extremity complications (DRLECs; exposure) or without DRLECs (comparator) that measured CI (outcome). DRLECs included DPN, diabetic foot ulcers, PAD, foot infections, and/or amputations in people with diabetes. Methodological quality of included studies assessed by the five-item Mixed Method Appraisal Tool. Heterogeneity assessed by I^2 statistic.	Original observational, experimental, and systematic review studies included that investigated CI and DRLECs in diabetic participants who did not have depression, anxiety, dementia, delirium, alcoholism, or neurodegenerative diseases. 1, 11, and 1 low, medium, and high methodological quality, respectively. 4 studies found CI differed in diabetes participants with vs. without DRLECs, 4 found significant associations of DRLECs to CI, 5 found no differences or associations. Meta-analysis of 3 eligible studies found CI did not differ significantly in participants with vs. without DPN, which held upon leave-one-out sensitivity analyses.	***Limitations***: Relatively few studies included that assessed DRLEC-CI relationship, most of low or medium quality and of small sample size; only 3 for meta-analysis, limiting interpretability. Studies exhibited heterogeneity, including in clinical (CI tools) and methodological (study design) aspects, potentially affecting results. Although excluded from the meta-analysis/systematic review, studies may still have included participants with depression. Search may not have identified all eligible studies, used a broad tool to assess methodological quality, and checked only half data extraction. ***Strengths***: None specifically stated.

Study	Country & region	Time*	Study participants	Study design	Study tools	Study findings	Study limitations/strengths**
**Liu et al.[11]**	United Kingdom	2006-2010 + 11.5 y follow-up	26,173 T2D participants	Longitudinal, population-based	UK Biobank study. Excluded participants without T2D or with stroke or dementia at baseline. DPN and retinopathy by self-report and hospital inpatient records. CKD further defined by eGFR or micro- or macro-albuminuria. Incident dementia and stroke by ICD-10 codes. Sociodemographic characteristics, lifestyle factors, medical history.	DPN linked to all-cause dementia (HR 1.93, 95%CI 1.34-2.86), AD (HR 1.95, 95%CI 1.08-3.51), VaD (HR 2.03, 95%CI 1.15-3.57), stroke (HR 1.48, 95%CI 1.05-2.07), and ischemic stroke (HR 1.47, 95%CI 1.02-2.12), but not hemorrhagic stroke (HR 1.27, 95%CI 0.62-2.60), adjusted for age, sex, ethnicity, T2D duration, HbA1c, BMI, LDL-c, SBP, drug use (antihyperglycemic, antihypertensive, statin), CHD history, presence of CKD and retinopathy, smoking, alcohol, exercise, diet, Townsend deprivation index. DPN linked most strongly to dementia though CKD and retinopathy also variably linked to dementia and stroke risk.	***Limitations***: Observational study, cannot fully exclude potential residual confounding. Some baseline microvascular disease by self-report. Outcomes by ICD codes, may have missed MCI cases not requiring hospitalization. Few incident cases by dementia subtypes, stronger link of microvascular disease to AD vs. VaD may be incidental. Uncertain generalizability. ***Strengths***: Large, prospective study with long-term follow-up. Wide range of traditional risk factors to fully adjust, health outcomes comprehensively captured with few participants lost to follow-up.
**Manschot et al.[12]**	Netherlands	Not stated	122 T2D participants, 56 matched controls	Cross-sectional, population-based case-control cohort	Utrecht Diabetic Encephalopathy Study. Excluded participants with psychiatric or neurological disorder (unrelated to T2D) that could influence CI, alcohol or substance abuse, or dementia. DPN by Toronto Clinical Neuropathy Scoring System, VPT, and a sensory examination sum score. CI by neuropsychological examination in 11 verbal and nonverbal tasks in 5 cognitive domains. WM lesions and cortical and subcortical atrophy by MRI. Metabolic variables.	Mean age (SD) 66.0 (5.8) y in T2D and 65.1 (5.2) y in controls; mean T2D duration (SD) 8.7 (6.1) y. PN present in 38% T2D participants and 12% of controls (p<0.001). T2D participants performed more poorly on neuropsychological tests, had more WM lesions (p<0.01) and atrophy (p<0.01) than controls. In T2D participants, no DPN measures linked to CI or MRI abnormalities in regression analyses, adjusted for age, sex, education.	***Limitations***: DPN measures used may be relatively less sensitive than brain functional and structural measures. Relation between central and peripheral neurological abnormalities might be more evident in participants with severe DPN. ***Strengths***: Detailed assessments of brain structure and function combined with different validated DPN and severity measures. Neurological examination per a standardized protocol. DPN prevalence in study like general population, potentially representative.
**Ming et al.[13]**	Germany	2020-2023	210 T2D participants, 51 T1D participants	Observational, single-center, cohort	DPN by NSS and NDS (either NDS≥6 or combined NDS≥3 plus NSS≥5); CI by MoCA. Game-based assessment of DPN and CI. Clinical variables.	DPN linked to CI (OR 1.87, 95%CI 1.06-3.36, p=0.03) in full cohort (n=261), adjusted for age, sex, weight, BMI, diabetes type, diabetes duration. DPN linked to CI (OR 1.95, 95%CI 1.03-3.76, p=0.04) in covariate-matched cohort (n=178), adjusted for age, sex, weight, BMI, diabetes type, diabetes duration. Predictive game features correlated with DPN (n=40), CI (n=59), or both (n=59). Models predicted with AUCs 0.95 for CI without DPN, 0.83 for DPN without CI, and 0.84 for both DPN and CI combined.	***Limitations***: Small sample size, especially T1D participants, so both T1D and T2D participants analyzed together introducing cohort heterogeneity. Lack of comprehensive DPN phenotyping so ability of gaming to detect DPN early debatable. Variable DPN manifestation. Fixed sequence of gaming tasks for standardization but repeated gaming might induce training effects, biasing results. Lack of familiarity of older demographic with gaming could affect performance. ***Strengths***: None specifically stated.
**Moreira et al.[14]**	Brazil	2011-2012	94 T2D participants, 54 controls	Cross-sectional, single-center, case-control cohort	Excluded participants for various criteria, including previous dementia, cerebroVD, or CAD diagnosis, cholinesterase inhibitor use, diagnosis of major psychiatric conditions, or any disease other than T2D linked to neuropathy. DPN by NSS and NDS; CI by MMSE, TMT-A, TMT-B, verbal fluency test (animals).	Mean age (range) 66.4 (60-75) y in T2D and 65.8 (61-84) y in controls. MMSE lower in T2D vs. controls (p <0.001), but not in other CI tests. No significant differences in any CI tests in T2D with vs. without DPN.	***Limitations***: Most participants female; DPN diagnosis based only in clinical scales and not nerve conduction tests; lacked data on variables that may impact DPN-CI association. DPN and CI may occur by differing mechanisms. ***Strengths***: None specifically stated.

Study	Country & region	Time*	Study participants	Study design	Study tools	Study findings	Study limitations/strengths**
**Palomo-Osuna et al.[15]**	Various countries	Included studies 1984-2021	T1D and T2D participants	Systematic review and meta-analysis, 19 studies	Included studies of T1D and T2D participants (population) with DPN (exposure) or without DPN (comparator) that measured CI (outcome). Methodological quality of included cross-sectional studies assessed by the Critical Appraisal Checklist for Analytical Cross-sectional Studies and of longitudinal studies by the Newcastle Ottawa Scale. Heterogeneity assessed by the DerSimonian and Laird method with Cochran's Q statistic.	Besides DPN and CI, examined factors such as depression, anxiety, cardiovascular risk, diabetes control, presence/intensity of pain. Included studies were of T1D and T2D participants who did not have diabetic autonomic neuropathy, preexisting psychiatric illness (e.g., schizophrenia), or neurological disease, e.g., Alzheimer's or Parkinson's disease. All included studies were of high methodological quality. DPN linked to global CI in T1D in 2 studies and T2D persons in 4 studies, as well as in specific domains. Meta-analysis of 4 eligible studies found CI significantly poorer in T2D participants with vs. without DPN (-1.0448, 95%CI -1.93% to -0.16%). Depression linked to CI (p<0.01).	***Limitations***: Relatively few studies with only 4 for meta-analysis. Variability in CI tools in different domains across various group comparisons, potentially affecting results. Search may not have identified all eligible studies.<br>***Strengths***: Study novelty.
**Putnam et al.[16]**	USA	2001-2018	184,765 T1D, matched to 524,602 T2D and 522,768 controls	Cross-sectional, population-based	Nationally representative healthcare claims database. Participants matched for age at study entry, sex, race/ ethnicity, geographic region, education, net worth, insurance type, high deductible health plan status, modified CCI, starting year of enrollment, follow-up duration. ICD-9/ICD-10 codes for T1D and T2D diagnosis, cognitive, mental health, addiction, chronic pain disorders, and microvascular (retinopathy, neuropathy, nephropathy) and macrovascular (stroke, MI, PVD) complications, amputation.	T1D (age 40-60 y: OR 0.89, 95%CI 0.85-0.94; age >60 y: OR 0.87, 95%CI 0.85-0.88) and T2D (age 40-60 y: OR 0.96, 95%CI 0.93-0.99; age >60 y: OR 0.89, 95%CI 0.88-0.90) linked to cognitive disorders vs. nondiabetic controls, adjusted for microvascular/ macrovascular complications. In all individuals, microvascular/macrovascular complications independently linked to cognitive disorders (age 40-60 y: OR 2.45, 95%CI 2.36-2.54; age >60 y: OR 2.16, 95%CI 2.13-2.19), adjusted for diabetes status. Neuropathy had largest effect size.	***Limitations***: Possible disease misclassification using ICD-9/ICD-10 codes, may have differentially captured severe neuropsychological outcomes prompting medical attention, claims data lacked detailed clinical data, e.g., complication, neuropsychological, diabetes severity. Uncertain generalizability.<br>***Strengths***: Large-scale claims data with wide range of neuropsychological outcomes across wide age range.
**Reynolds et al.[17]**	Gila River Community, USA	2017-2019	51 T2D participants	Cross-sectional, single-center	Pima American Indians from the Gila River Community. DPN by MNSI; CI by NIHTB-CB; brain imaging by structural/functional MRI; metabolic parameters, diabetic complications.	Cohort mean (SD) age 48.4 (11.3) y, T2D duration 20.1 (9.1) y. Participants had similar NIHTB-CB composite score vs. normative data. T2D duration, but no other metabolic risk factors, linked to lower cortical thickness and GM volume and higher WMH volume. CAN and CKD linked to lower GM volume.	***Limitations***: Cross-sectional, small study, lacked nondiabetic control group, uncertain generalizability.<br>***Strengths***: None specifically stated.
**Rhmari Tlemçani et al.[18]**	Morocco	2021-2022	100 T2D participants	Cross-sectional, single-center, cohort	Excluded participants with history of ischemic stroke and depression, use of antiepileptic drugs, HbA1c< 4%, older >75 y. DPN by MNSI; CI by MMSE. Clinical variables, comorbidities (hyperuricemia, hypertension, amputation, cardioVD), microvascular (retinopathy, nephropathy) and macrovascular complications (CHD, PAD).	Median age (IQR) 65 (59-70) y, T2D duration15 (9-20) y. DPN not significantly more prevalent in T2D participants with CI. MMSE score linked to older age (β -0.0975; 95%CI -0.163 to -0.00324, p=0.004), retinopathy (β -1.825, 95%CI -3.109 to -0.541, p<0.001), dyslipidemia (β -3.423, 95%CI -4.793 to -2.054, p<0.001), adjustments not stated.	***Limitations***: Sample size; potential selection bias since study at tertiary center; lacked data on diabetes, hypertension, and dyslipidemia medication use; used creatinine instead of glomerular filtration rate for CKD.<br>***Strengths***: Well-phenotyped MCI participants; used neuropsychological tests standardized for the Moroccan population.

Study	Country & region	Time*	Study participants	Study design	Study tools	Study findings	Study limitations/strengths**
**Satapathy et al.[19]**	Various countries	Included studies 2012-2023	T2D participants	Meta-analysis, 7 studies	Included studies of T2D participants (population) with diabetic complications (exposure) or without diabetic complications (comparator) that measured CI (outcome). Methodological quality of included studies assessed by Methodological Index for Non-Randomized Studies. Heterogeneity assessed by I^2 statistic.	Besides diabetic complications and CI, examined depression. CI prevalence 34.9, 55.29, 40.97% in patients with DPN, retinopathy, nephropathy, respectively. Depression prevalence 43.30, 40.17, 18.92% in patients with DPN, retinopathy, nephropathy, respectively. CI linked to nephropathy (OR 1.421, 95%CI 1.086-1.858) but not DPN (OR 0.878, 95%CI 0.298-2.585) or retinopathy (OR 1.358, 95%CI 0.957-1.927). Depression not linked to any complication.	***Limitations***: Few included studies and participants, mostly cross-sectional, cannot assess causality. Could not fully address possible confounding. Variability in diagnostic criteria, definitions, participant characteristics potentially affecting results. Publication bias not fully assessed due to few included studies. Restricted to English-language studies. ***Strengths***: Registered protocol, comprehensive literature searches of multiple databases, independent dual screening and data extraction, formal quality appraisal.
**Selvarajah et al.[20]**	United Kingdom	2009-2019	T1D and T2D participants without DPN (n=57), with painful DPN (n=77), with painless DPN (n=77), age- and sex-matched controls (n=66)	Cross-sectional, observational, single-center, case-control cohort	See Selvarajah et al.[21] for exclusion criteria. DPN by NCS (American Academy of Neurology minimum case definition criteria), painful DPN (International Association for the Study of Pain definition) and DN4≥4. Sensory phenotyping by QST (German Research Network on Neuropathic Pain protocol) to classify painful DPN as irritable (IR) vs. nonirritable (NIR) nociceptor phenotypes. Clinical history, physical examination. Brain volume by MRI.	Primary somatosensory and motor cortical thickness reduced in painful and painless DPN participants vs. controls (p=0.02) and participants without DPN (p=0.01). Somatomotor cortical thickness linked to DPN severity. Ventrobasal thalamic nuclei volume reduced in both painless and painful DPN. Reduced primary somatosensory cortical, posterior cingulate cortical, and thalamic volume in painful DPN participants with NIR vs. IR nociceptor phenotypes. Analyses adjusted for age and sex.	***Limitations***: Cross-sectional, cannot assess causality. Sex imbalance across groups but were adjusted in MRI analyses. Coexisting mood disorders not considered. Focused on brain regions mainly involved in somatosensory and nociceptive processing but should also examine other regions involved in wider pain processing. Pain phenotype classification mostly relied on superficial and not deep sensory function. Potential overlap of pain phenotypes, unusually high proportion of painful DPN participants with IR nociceptor phenotype. ***Strengths***: Participants were well characterized.
**Shapiro et al.[22]**	United States	2002-2019	T1D (n=646) and T2D (n=165) participants	Longitudinal, population-based	SEARCH for Diabetes in Youth Study. DPN by MNSI; CI by NIHTB-CB; other diabetic microvascular complications (retinopathy, nephropathy); clinical and metabolic factors. Aged-adjusted clustering of variables (including DPN) with CI outcomes.	**T1D**: mean (SD) age 21.7 (4.2) y, T1D duration 11.1 (3.3) y at 2nd follow-up. Lowest composite and individual fluid and crystallized cognitive scores clustered with DPN and self-reported hypoglycemic events in the year preceding CI testing. **T2D**: mean (SD) age 24.5 (4.4) y, T2D duration 9.9 (3.6) y at 2nd follow-up. Lowest composite and individual fluid and crystallized cognitive scores clustered with DPN, higher waist-to-height ratio area under the curve, higher SBP, higher central SBP and DBP, depression.	***Limitations***: Did not collect baseline cognition (precluded analysis of CI changes to diabetes development), functional outcomes (e.g., academic performance to verify NIHTB-CB scores), or consider medication use (results only interpretable independent of potential medication effects on CI). ***Strengths***: Investigated co-occurrence of diabetic complications, clinical risk factors, and CI outcomes in youth-onset diabetes
**Yen et al.[23]**	Taiwan	2009-2014	Newly diagnosed T2D participants score-matched with (n=79,241)/ without (n=568,577) microvascular complications	Retrospective, population-based	National Health Insurance Research Database. Excluded participants with T1D, microvascular complications prior to T2D diagnosis, prior CI or memory loss. ICD-9/ICD-10 codes to identify microvascular (diabetic neuropathy, retinopathy, nephropathy) complications and outcomes (AD, VaD, other dementias [Pick's disease, senile brain degeneration, dementia with Lewy bodies]). Clinical variables, medication use.	Mean 6.54 y follow-up. Diabetic neuropathy linked to AD (HR 1.14, 95%CI 1.03-1.27, p=0.01), VaD (HR 1.14, 95%CI 1.02-1.28, p=0.02), other dementias (HR 1.10, 95%CI 1.04-1.16, p<0.001), and all-cause dementia (HR 1.12, 95%CI 1.06-1.17, p<0.001), adjusted for age, sex, obesity, smoking, alcohol-related disorders, co-morbidities, antidiabetic and cardiovascular-related medication use.	***Limitations***: Retrospective, cannot exclude unknown residual confounding. Lacked data on clinical, laboratory, diet, exercise, alcohol use, family history. All participants diagnosed with CKD within 1 year of T2D diagnosis labeled as DKD, though other causes possible. Most participants of Chinese descent, uncertain generalizability. ***Strengths***: Baseline demographics, clinical variables, and medication use balanced between participants with various microvascular complications.

Study	Country & region	Time*	Study participants	Study design	Study tools	Study findings	Study limitations/strengths**
**Zhao et al.[24]**	China	Not stated	T2D participants without DPN (n=20), subclinical DPN (n=12), with DPN (n=23)	Cross-sectional, single-center, cohort	Excluded participants with CNS conditions (e.g., cerebroVD, retinopathy, psychiatric diseases, brain trauma, tumors, WM defects), history of CNS surgery, history of alcohol or drug abuse. DPN by NSS, NDS, DN4, NCS, QST, classified into participants without DPN, subclinical DPN, and with DPN based on clinical symptoms and neurophysiological assessments. Brain morphology and function by MRI. Clinical variables.	Orbitofrontal to bilateral postcentral and middle temporal cortex functional connectivity increased in T2D participants with subclinical vs. without DPN. Orbitofrontal GM volume and functional connectivity transiently increased in T2D participants with subclinical vs. with DPN. Orbitofrontal cortex GM volume negatively linked to NSS, NDS, and DN4 and positively linked to bilateral peroneal amplitude and right sensory sural NCVs. Orbitofrontal to postcentral cortex functional connectivity positively linked to cold TPT and negatively linked to NSS.	***Limitations***: Relatively small study; cross-sectional, cannot assess causality. Lacked tests of CI so correlation to brain changes not assessed. ***Strengths***: Included T2D participants with subclinical DPN to assess relation to early brain changes.

95%CI, 95% confidence interval; ACE, angiotensin converting enzyme; ACR, albumin-creatine ratio; AD, Alzheimer's disease; APOE4, apolipoprotein E4; AUC, area under the curve; BMI, body mass index; BP, blood pressure; CAN, cardiac autonomic neuropathy; CAD, coronary artery disease; cardioVD, cardiovascular disease; CCI, Charlson Comorbidity Index; cerebroVD, cerebrovascular disease; CHD, coronary heart disease; CI, cognitive impairment; CKD, chronic kidney disease; CMAP, compound motor action potential; CRP, C-reactive protein; CSF, cerebrospinal fluid; DBP, diastolic blood pressure; DKD, diabetic kidney disease; DN4, Douleur Neuropathique en 4 Questions; DPN, diabetic peripheral neuropathy; DSST, Digit Symbol Substitution Test; eGFR, estimated glomerular filtration rate; EMG, electromyography; EURODIAB PCS, EURODIAB Prospective Complications Study risk score; GM, grey matter; HbA1c, hemoglobin A1c; HDL-c, high-density lipoprotein cholesterol; HR, hazard ratio; ICD, International Classification of Diseases; IQR, interquartile range; LANSS, Leeds Assessment of Neuropathic Symptoms and Signs; LDL-c, low-density lipoprotein cholesterol; LEA, lower extremity amputation; MCI, mild cognitive impairment; MI, myocardial infarction; MMSE, Mini-Mental State Examination; MNSI, Michigan Neuropathy Screening Instrument; mo, months; MoCA, Montreal Cognitive Assessment; MRI, magnetic resonance imaging; NCS, nerve conduction study; NCV, nerve conduction velocity; NDS, Neuropathy Disability Score; NIHTB-CB, NIH Toolbox Cognition Battery; NIS, Neuropathy Impairment Score; NSS, Neuropathy Symptom Score; NTSS-6, Neuropathy Total Symptom Score-6; OR, odds ratio; PAD, peripheral artery disease; PN, peripheral neuropathy; PVD, peripheral vascular disease; QST, quantitative sensory testing; SBP, systolic blood pressure; SEVLT, Spanish English Verbal Learning Test; SD, standard deviation; SES, socioeconomic status; SPMSQ, Short Portable Mental Status Questionnaire; T1D, type 1 diabetes; T2D, type 2 diabetes; TMT, Trail Making Test; TPT, thermal perception threshold; TSH, thyroid-stimulating hormone; WAIS, Wechsler Adult Intelligence Scale; WC, waist circumference; WHO, World Health Organization; WM, white matter; WMH, white matter hyperintensity; VaD, vascular dementia; VPT, vibration perception threshold; y, year.

References

1. Barzilay JI, Ghosh A, Busui RP, et al. The cross-sectional association of cognition with diabetic peripheral and autonomic neuropathy-The GRADE study. *J Diabetes Complications*. Dec 2021;35(12):108047. doi:10.1016/j.jdiacomp.2021.108047
2. Brenowitz WD, Robbins NM, Strotmeyer ES, Yaffe K. Associations of Lower Extremity Peripheral Nerve Impairment and Risk of Dementia in Black and White Older Adults. *Neurology*. May 3 2022;98(18):e1837-e1845. doi:10.1212/wnl.0000000000200185
3. Chang PY, Wang, II, Chiang CE, et al. Vascular complications of diabetes: natural history and corresponding risks of dementia in a national cohort of adults with diabetes. *Acta Diabetol*. Jul 2021;58(7):859-867. doi:10.1007/s00592-021-01685-y
4. de Bresser J, Reijmer YD, van den Berg E, et al. Microvascular determinants of cognitive decline and brain volume change in elderly patients with type 2 diabetes. *Dement Geriatr Cogn Disord*. 2010;30(5):381-6. doi:10.1159/000321354
5. Ding X, Fang C, Li X, et al. Type 1 diabetes-associated cognitive impairment and diabetic peripheral neuropathy in Chinese adults: results from a prospective cross-sectional study. *BMC Endocr Disord*. Mar 27 2019;19(1):34. doi:10.1186/s12902-019-0359-2
6. Elsharkawy RE, Abdel Azim GS, Osman MA, et al. Peripheral Polyneuropathy and Cognitive Impairment in Type II Diabetes Mellitus. *Neuropsychiatr Dis Treat*. 2021;17:627-635. doi:10.2147/ndt.s284308
7. Ferik S, Güven H, Ateş MP, Conkbayır I, Çomoğlu S, Güven B. Diabetic polyneuropathy, deep white matter lesions, and carotid atherosclerosis: is there any association? *Neurol Sci*. Jan 2018;39(1):103-110. doi:10.1007/s10072-017-3160-x
8. Hansen TM, Muthulingam JA, Brock B, et al. Reduced gray matter brain volume and cortical thickness in adults with type 1 diabetes and neuropathy. *Neurosci Res*. Mar 2022;176:66-72. doi:10.1016/j.neures.2021.10.002
9. Hicks CW, Wang D, Schneider ALC, et al. Associations of Peripheral Neuropathy Defined by Monofilament Insensitivity with Mild Cognitive Impairment and Dementia in Older Adults. *Dement Geriatr Cogn Disord*. 2022;51(2):150-158. doi:10.1159/000523762
10. Karunathilaka N, Parker C, Lazzarini PA, et al. Cognitive changes in people with diabetes with lower extremity complications compared to people with diabetes without lower extremity complications: a systematic review and meta-analysis. *BMC Endocr Disord*. Nov 29 2024;24(1):258. doi:10.1186/s12902-024-01774-3
11. Liu YJ, Zhao JY, Han WW, et al. Microvascular burden and long-term risk of stroke and dementia in type 2 diabetes mellitus. *J Affect Disord*. Jun 1 2024;354:68-74. doi:10.1016/j.jad.2024.03.053
12. Manschot SM, Biessels GJ, Rutten GE, Kessels RP, Gispen WH, Kappelle LJ. Peripheral and central neurologic complications in type 2 diabetes mellitus: no association in individual patients. *J Neurol Sci*. Jan 15 2008;264(1-2):157-62. doi:10.1016/j.jns.2007.08.011

13. Ming A, Lorek E, Wall J, et al. Unveiling peripheral neuropathy and cognitive dysfunction in diabetes: an observational and proof-of-concept study with video games and sensor-equipped insoles. *Front Endocrinol (Lausanne)*. 2024;15:1310152. doi:10.3389/fendo.2024.1310152

14. Moreira RO, Soldera AL, Cury B, Meireles C, Kupfer R. Is cognitive impairment associated with the presence and severity of peripheral neuropathy in patients with type 2 diabetes mellitus? *Diabetol Metab Syndr*. 2015;7:51. doi:10.1186/s13098-015-0045-0

15. Palomo-Osuna J, De Sola H, Dueñas M, Moral-Munoz JA, Failde I. Cognitive function in diabetic persons with peripheral neuropathy: a systematic review and meta-analysis. *Expert Rev Neurother*. Mar 2022;22(3):269-281. doi:10.1080/14737175.2022.2048649

16. Putnam NM, Reynolds EL, Banerjee M, et al. Neuropsychological Outcomes in Individuals With Type 1 and Type 2 Diabetes. *Front Endocrinol (Lausanne)*. 2022;13:834978. doi:10.3389/fendo.2022.834978

17. Reynolds EL, Votruba K, Jack CR, et al. Association between brain health outcomes and metabolic risk factors in persons with diabetes. *Ann Clin Transl Neurol*. Oct 2023;10(10):1891-1898. doi:10.1002/acn3.51859

18. Rhmari Tlemçani FZ, Elamari S, Motaib I, et al. Factors Associated With Mild Cognitive Impairment in Patients With Type 2 Diabetes: A Cohort Study. *Cureus*. Aug 2022;14(8):e28305. doi:10.7759/cureus.28305

19. Satapathy P, Pratima P, Gaidhane AM, et al. Prevalence and impact of microvascular complications in type 2 diabetes mellitus on cognitive impairment and depression: a systematic review and meta-analysis. *Diabetol Metab Syndr*. Jun 3 2025;17(1):187. doi:10.1186/s13098-025-01759-9

20. Selvarajah D, Sloan G, Teh K, et al. Structural Brain Alterations in Key Somatosensory and Nociceptive Regions in Diabetic Peripheral Neuropathy. *Diabetes Care*. Apr 1 2023;46(4):777-785. doi:10.2337/dc22-1123

21. Selvarajah D, Wilkinson ID, Maxwell M, et al. Magnetic resonance neuroimaging study of brain structural differences in diabetic peripheral neuropathy. *Diabetes Care*. Jun 2014;37(6):1681-8. doi:10.2337/dc13-2610

22. Shapiro ALB, Bellatorre A, Dabelea D, et al. Diabetes complications and cognitive function in young adults with youth-onset type 1 or type 2 diabetes: the SEARCH for Diabetes in Youth Study. *Pediatr Diabetes*. 2023;2023doi:10.1155/2023/4860831

23. Yen YH, Yen FS, Ko FS, et al. Microvascular disease and its association with dementia in patients with type 2 diabetes: A nationwide cohort study in Taiwan. *Diabetes Obes Metab*. Nov 2024;26(11):5399-5407. doi:10.1111/dom.15908

24. Zhao LM, Chen X, Zhang YM, et al. Changed cerebral function and morphology serve as neuroimaging evidence for subclinical type 2 diabetic polyneuropathy. *Front Endocrinol (Lausanne)*. 2022;13:1069437. doi:10.3389/fendo.2022.1069437
